# The modulation of stomatal conductance and photosynthetic parameters is involved in Fusarium head blight resistance in wheat

**DOI:** 10.1371/journal.pone.0235482

**Published:** 2020-06-30

**Authors:** Sara Francesconi, Giorgio Mariano Balestra

**Affiliations:** Dipartimento di Scienze Agrarie e Forestali (DAFNE), Università degli Studi della Tuscia, Viterbo, Italy; Institute of Genetics and Developmental Biology Chinese Academy of Sciences, CHINA

## Abstract

Fusarium head blight (FHB) is one of the most devastating fungal diseases affecting grain crops and *Fusarium graminearum* is the most aggressive causal species. Several evidences shown that stomatal closure is involved in the first line of defence against plant pathogens. However, there is very little evidence to show that photosynthetic parameters change in inoculated plants. The aim of the present study was to study the role of stomatal regulation in wheat after *F*. *graminearum* inoculation and explore its possible involvement in FHB resistance. RT-*q*PCR revealed that genes involved in stomatal regulation are induced in the resistant Sumai3 cultivar but not in the susceptible Rebelde cultivar. Seven genes involved in the positive regulation of stomatal closure were up-regulated in Sumai3, but it is most likely, that two genes, *TaBG* and *TaCYP450*, involved in the negative regulation of stomatal closure, were strongly induced, suggesting that FHB response is linked to cross-talk between the genes promoting and inhibiting stomatal closure. Increasing temperature of spikes in the wheat genotypes and a decrease in photosynthetic efficiency in Rebelde but not in Sumai3, were observed, confirming the hypothesis that photosynthetic parameters are related to FHB resistance.

## Introduction

Stomatal conductance is the physiological plant mechanism needed for gas exchange in order to achieve an optimal photosynthetic process by adjusting water transpiration. The movements of stomata occur through the bumping and lessening of the guard cells and stomatal closure is the primary response of plants to water deficit. Abscisic acid (ABA), jasmonate (JA), ethylene, auxins and cytokinins interact in a composite network of cellular signals as modulators of stomatal closure. ABA functionality is strongly dependent upon synchronized activation of biosynthetic, catabolic, conjugating/deconjugating and transporting genes [1]. The positive regulation of stomatal closure starts in the plastids, where the first activated enzyme is the terpene synthase (TPS). During the following step the zeaxanthin epoxidase (ZEP) catalyses the conversion of zeaxanthin and antheraxanthin into violaxanthin. Violaxanthin is transformed into 9-cis-epoxycarotenoid by 9-cis-epoxycarotenoid dioxygenase (NCED). Xanthonin is transferred to the cytoplasm, where ABA-aldehyde is generated and the ABA-aldehyde oxidase (AAO) catalyses its conversion into ABA. The adjustment of stomatal movements requires also the activation of negative regulators, such as the cytochrome P450 family (CYP450) and the β-1,3-glucanase (BG), which can deconjugate or conjugate ABA. In the guard cells, phaseic acid binds the ABA receptor (REC) forming a complex that inhibits phosphatase (ABI). The inactivation of ABI permits the activation of downstream targets [1–4]. The signalling cascade begins with the activation of different kinases, such as Ca^2+^-dependent protein kinases (CDPKs) and mitogen-activated protein kinases (MAPKs). Moreover, the guard cells produce reactive oxygen species (ROS), for instance hydrogen peroxide (H_2_O_2_) and nitric oxide (NO). NADPH oxidase (RBOH) facilitates the generation of ROS in the stomata, which results to stomatal closure. CDPKs are linked to the JA-signalling pathway, which is positively implicated in stomatal closure. JA is subjected to metabolism by allene oxide synthase (AOS) and hydroperoxide lyase (HPL). The resulting signalling cascade activates several response-genes thanks to MYC and MYB domain transcription factors. [5–7].

Plants are incessantly subjected to an evolutionary pressure by the attack of diverse pathogens. Hence, plant physiology has evolved several defence strategies to counteract plant pathogens [8]. Plants evolved a specific type of recognition receptors, called pattern recognition receptors (PRRs), which are able to recognize conserved-elicitor domains, the pathogen-associated molecular patterns (PAMPs) [8]. Perception of PAMPs drives those basal defence responses, the PAMP-triggered immunity (PTI), which activates several physiological defence mechanism: MAPK cascades, alkalinisation of the extracellular liquor, ROS and NO production, increases in Ca^2+^ influx, synthesis of salicylic acid (SA), ethylene, and induction of stomatal closure [9]. A wide range of foliar pathogens are known to disrupt stomatal movements in the presence of infection. Studies have shown that stomata are not just a passive gate for pathogen entry but play an active role in the plant innate immune response, and the control of stomatal closure is one of the first lines of defence against pathogen invasion. Therefore, plants can restrict pathogen entry into the leaves by closing stomata or by inhibiting stomatal opening. Conversely, pathogens have evolved virulence factors to counteract host stomatal defences by inhibiting stomatal closure or promoting stomatal opening. In this regard, the physiological regulation of stomatal closure has a critical function during the plant-pathogen interaction, acting as a prompt barrier against invasion by pathogens [10–12].

Among the fungal plant diseases, Fusarium head blight (FHB) of wheat (*Triticum aestivum* L. and *T*. *turgidum* subsp. *durum* (Desfontaines) Husnache), barley (*Hordeum vulgare* L.) and other cereals is one of the most devastating for grain crops [13]. *Fusarium graminearum* Schwabe is the predominant species which causes FHB in many countries [14]. The wheat florets represent the first infection site for the fungal spores, which infect the head at anthesis, especially at warm and humid environmental conditions [15]. Afterwards, the hypha of *F*. *graminearum* proliferates and invades the host tissues predominantly by direct penetration and passive entry, where plant stomata has a crucial role, because through them the fungus infects the spikelets internally by entering into the vascular bundles of the rachilla and rachis [16]. Spores germinate and initiate infection, generating typical symptoms, such as dark-brown, water-soaked spots on the infected spikes which bleach completely [17]. FHB is an extremely problematic disease because these pathogens produce mycotoxin deoxynivalenol (DON), toxic to human and animal safety [18]. Resistance to FHB in wheat involves active and passive mechanisms, such as cell reinforcement, hypersensitive reaction (HR), production of phytoalexins and induction of defence-related genes [19]. Resistance to FHB is quantitative and multigenic although the genetic function of many QTLs is still unknown [20]. Many studies support the hypothesis that resistance to FHB is capable of arousing different signalling cascades in the host plant, following recognition of pathogens, such as Ca^2+^ efflux, activation of JA and MeJA related genes, ROS production and activation of systemic-acquired resistance (SAR) [21,22]. Additionally, few studies have highlighted the idea that there is phenotypic evidence that photosynthetic parameters change in order to counteract different fungal pathogens [23].

The genetic mechanism of the regulation in wheat of stomatal conductance to FHB response remains still unclear and its elucidation would provide new information valuable to breeding programmes. In the present work we selected the main genes involved in stomatal closure in order to evaluate their response to FHB. The relative expression levels of the susceptible Italian bread wheat cultivar, Rebelde, and the experimental resistant Sumai3 genotype bread wheat were compared. Furthermore, the relative expression gene levels of inoculated and drought-stressed plants were compared, to detect the existence of a putative differential gene response between FHB and water stress. Additionally, spike temperature, photosynthetic efficiency and morphology of stomata were evaluated as direct phenotypical parameters.

## Materials and methods

### Fungal material and preparation of the inoculum

The highly virulent and DON-producing isolate of *F*. *graminearum* wild type 3824 was isolated for the first time by the University of Pisa [24]. The isolate was cultured at 21°C on Synthetic Nutrient Poor Agar (SNA) to obtain macroconidia [25]. After 10 days on SNA, the conidia were scraped with a glass rod after pipetting 1 mL of sterile distilled water onto the surface of a Petri dish. The conidial suspension was recovered, and the concentration adjusted to 1x10^5^ conidia mL^-1^ using a Thoma Chamber (0.100 mm depth and 0.0025 mm^2^). The inoculum was prepared in sterile distilled water supplemented with 0.05% (v/v) of Tween-20.

### Plant material, growth and inoculation conditions and experimental design

The FHB-susceptible Italian wheat Rebelde and the resistant Sumai3 (bread wheat) were grown in the greenhouse. The surfaces of the kernels were sterilized with sodium hypochlorite (0.5% v/v) for 20 minutes and then rinsed twice for 5 minutes in sterile distilled water. The kernels were germinated in the dark on paper soaked in sterile distilled water for 15 days at 4°C to break dormancy, followed by 2 days at room temperature. The seedlings were transferred to 40×20 cm pots, filled with TYPical Brill soil and grown at 16°C–20°C up to the boot stage, 20°C-24°C during anthesis and 24°C–29°C up to maturity. The plants were fertilised using ammonium nitrate in the following proportions and at the following stages: 20% at sowing, 40% at tillering and 40% at heading [26]. The plants were subjected to three different treatments: i) drought stress (the plants did not receive water from the boot (Zadok stage 51) to flowering stages (Zadok stage 69) [27,28]); ii) artificial inoculation, (the plants were spray-inoculated at the flowering stage by applying a suspension of 100 μL of 1x10^5^ conidia mL^-1^ in the central floret by using a manual nasal sprayer; the artificial inoculation lasted 10 days and the phenological stage of the plants was checked day by day, in order to inoculate at Zadok stage 69 [26,29]); iii) mock, (at the flowering stage the central floret was sprayed with 100 μL of Tween-20 0.05% resuspended in sterile distilled water). The spikes were homogenously sprayed with sterile distilled water and covered with clear plastic bags for 24 hours to maintain high humidity levels (>80%). The spikes subjected to drought and mock treatments were sampled after removing the plastic bags, while the inoculated spikes were sampled 24, 48, 72 hours post inoculation (hpi) and 10 days post inoculation (dpi) to investigate an early or a late response to *F*. *graminearum*. The spikes collected were immediately stored in liquid nitrogen and at -80°C until the extraction of RNA. The disease severity of the FHB (%) was determined by counting the number of bleached spikelets for each inoculated spike from 3 to 21 dpi (26). Data were obtained from three independent replicates with each replicate consisting of 20 spikes for each treatment.

### Identification of *in-silico* sequences, homology and phylogenetic analysis and primer design

Sequence-similarity searches were performed using the URGI BLAST Database Wheat Genome (IWGSC_ref_v1) (https://wheat-urgi.versailles.inra.fr/). The similarity searches were carried out starting from known sequences of *Aegilops tauschii* Cosson, *H*. *vulgare*, *Oryza sativa* L. and *Zea mays* L. obtained from Gramene (www.gramene.org). Homologue sequences were extracted and aligned by using CLUSTUL MUSCLE (v3.8.31) configured for highest accuracy. Primers were designed to amplify the entire nucleotide sequences from the Rebelde and Sumai3 samples. Homology and phylogeny trees were generated using DNAMAN software (Lynnon Biosoft, Quebec, Canada) using the maximum-likelihood method and setting the bootstrap values from a minimum of 1000 trials. The sequences were submitted to BLASTn (https://blast.ncbi.nlm.nih.gov/) analysis in order to extract the corresponding cDNA. Primers for Real-Time *q*PCR were designed inside the exonic regions. All the primers were designed using Primer3Plus 2.4.0. Primers for Glyceraldehyde-3-phosphate dehydrogenase *(TaGAPDH)*, Pathogenesis Related Protein 1(*TaPR1)*, Actin, (*TaACT)*, Tubulin (*TaTUB)* and Ferredoxin-NADP(H)-oxidoreductase (*TaFNR*) have been taken from the following literature references: *TaPR1* [30], *TaACT* [31], *TaTUB*, *TaFNR* [32] and *TaGAPDH* [33]. The primer pair for *TaGAPDH* amplification has been adapted for *T*. *aestivum* since they have been originally designed for *H*. *vulgare* sequence. S1 Table shows the list of selected genes, their functions, the corresponding primer pairs and the amplicon lengths for the DNA and cDNA, while S2 Table reports the accession numbers of homologous sequences used for sequence-similarity searches.

### DNA extraction and PCR

Wheat DNA was extracted from mature kernels of Rebelde and Sumai3. The kernels were ground to obtain a fine powder and 10 mg were subjected to DNA extraction using an extraction buffer composed of Tris 100 mM, EDTA 50 mM and NaCl 500 mM, SDS 10% (w/v), potassium acetate 5 M. The DNA was resuspended in 20 μL of DNase and RNase-free sterile distilled water and stored at -20°C. Total DNA was quantified with Qubit™ fluorometer 1.01 (Invitrogen) using the Qubit™ dsDNA BR Assay Kit (Thermo Fisher Scientific) and diluted to 10 ng μL^-1^. A gradient PCR was performed by following the instruction of GoTaqGreen MasterMix (Promega). The PCR was prepared in a total volume of 10 μL and conditions of amplification included: an initial denaturation step of 2 minutes at 95°C; 35 cycles of 30 seconds denaturation at 95°C; 40 seconds of annealing at 55–65°C; 60 seconds of elongation at 72°C; a final elongation step of 5 minutes at 72°C. Once the optimal annealing temperature (60°C) was obtained, the sequences were amplified following the PCR conditions described previously. The amplicon unicity was visualized on 1.5% agarose gel and confirmed by Sanger sequencing (Eurofins Genomics).

### RNA extraction and cDNA synthesis

The RNA was extracted from entire wheat heads. Each head was ground in liquid nitrogen using a mortar and pestle until a fine powder was obtained. 100 mg of powder was subjected to RNA extraction following the instructions provided by InviTrap® Spin Plant RNA Mini Kit (Stratec Molecular). The RNA was resuspended in RNase-free sterile distilled water and immediately poured onto ice and quantified with Qubit™ fluorometer 1.01 (Invitrogen) using the Qubit™ RNA BR Assay Kit (Thermo Fisher Scientific). To confirm the total quality and integrity of the RNA, 5 μL of the extracted RNA was subjected to thermal shock (10 minutes at -80°C after 5 minutes at 65°C) and run on a 1.5% denaturating agarose gel. The synthesis of the cDNA was performed using 500 ng of RNA following the instructions provided by Xpert cDNA Synthesis Supermix with a gDNA eraser (GRiSP Research Solutions) in a final volume of 20 μL. To ensure that the synthesis of the cDNA and the elimination of the gDNA had succeeded, a reverse transcriptase PCR (RT-PCR) of *TaACT* (containing an intron in the sequence amplified by the primer pair selected) was performed. The RT-PCR was performed by following the instruction provided by GoTaqGreen MasterMix (Promega) in a total volume of 10 μL and conditions of amplification included: an initial denaturation step of 2 minutes at 95°C; 35 cycles of 30 seconds denaturation at 95°C; 40 seconds of annealing at 60°C; 30 seconds of elongation at 72°C; a final elongation step of 5 minutes at 72°C. The amplification run consisted also of a no-template control (NTC) and a genomic DNA (gDNA) control. The amplicons were visualised on a 1.5% agarose gel.

### RT-*q*PCR

The efficiency of the RT-*q*PCR amplification (E) was determined for each primer pair and for each wheat genotype as follows: five 1:10 serial dilutions (1:1–1:10000) were obtained for each cDNA and amplified in four replicates. E and correlation coefficient (R^2^) values were calculated by means of the slope of the standard curve obtained by plotting the fluorescence versus the serial dilution concentrations using the equation $E=10^{\left(-\frac{1}{slope}\right)}-1$. The most stable reference gene was chosen on the basis of the E value most similar to the E values of target genes, the highest R^2^ and the lowest variability for the quantification cycles (Cq). The relative expression levels of the target genes were calculated on the basis of the Cq values of the four technical replicates derived from four independent biological replicates for each plant treatment by applying the equation *Relative expression* = 2^−ΔΔ*Cq*^ using *TaACT* as the reference gene and the mock treatment to normalize the relative expression levels. The relative expression levels of *TaPR1* and *TaGAPDH* were quantified as internal control of the progression of the infection [34] and changes in photosynthesis [35]. The RT-*q*PCR was performed, following the instructions provided by the Rotor Gene Q (Qiagen) and Xpert Fast SYBR (uni) MasterMix (Grisp), in a final volume of 10 μL. The amplification conditions included an initial denaturation step of 3 minutes at 95°C; 40 cycles of 5 seconds denaturation at 95°C; 30 seconds of annealing at 60°C and 20 seconds of elongation at 72°C. A final melt cycle (70–99°C) and a Sanger sequencing of the amplified cDNAs were performed to confirm the amplicons’ unicity and identity. NTC controls were included and the amplification was considered negative when a value of Cq ≥ 38 was detected [36].

### The spike-temperature and photosynthetic-efficiency measurements and optical microscopy

Spike-temperature and photosynthetic efficiency were measured as phenotypical parameters of stomatal regulation. The spike temperature was recorded using a portable thermometer (Extech 1200F Compact Laser Infrared Thermometer 42510A), while the photosynthetic efficiency was measured using a portable fluorometer (V2.00f PAM 2000). Phenotypical parameters were measured on three independent replicates each one comprising of 20 individual spikes for each treatment and inoculation time-point. Each measurement was carried out at dawn. At the same time, three external glumes were randomly collected from the spikes for each treatment and inoculation time-point. A thin portion of the glumes was obtained and analysed with an optical microscope (Leitz Diaplam) using a 40X magnification in order to observe the stomata.

### Statistical analyses

Data from the RT-*q*PCR were subjected to two-way variance analysis (ANOVA), where the two independent variables were the wheat genotype (Rebelde and Sumai3) and the treatments undergone by the plants (drought stress, 24 hpi, 48 hpi, 72 hpi and 10 dpi) while the dependent variable was the relative expression level. FHB disease severity and phenotypical data were subjected to one-way ANOVA analysis. Two levels of significance (*P*<0.05 and *P*<0.01) were computed to assess the significance of the F values. A pairwise analysis was carried out using the Tukey Honestly Significant Difference test (Tukey test) at the 0.95 or 0.99 confidence level. The Pearson’s linear correlation was computed to establish the correlation between the genotypes and the photosynthetic parameters. Statistical analyses were performed using SYSTAT12 software (Systat Software Inc., San Jose, CA, U.S.A.). Principal component analysis (PCA) and heatmap were carried out using ClustVis software [37].

## Results

### Evaluation of FHB severity in the susceptible bread wheat genotype Rebelde and in the resistant bread wheat Sumai3

**Fig 1:**
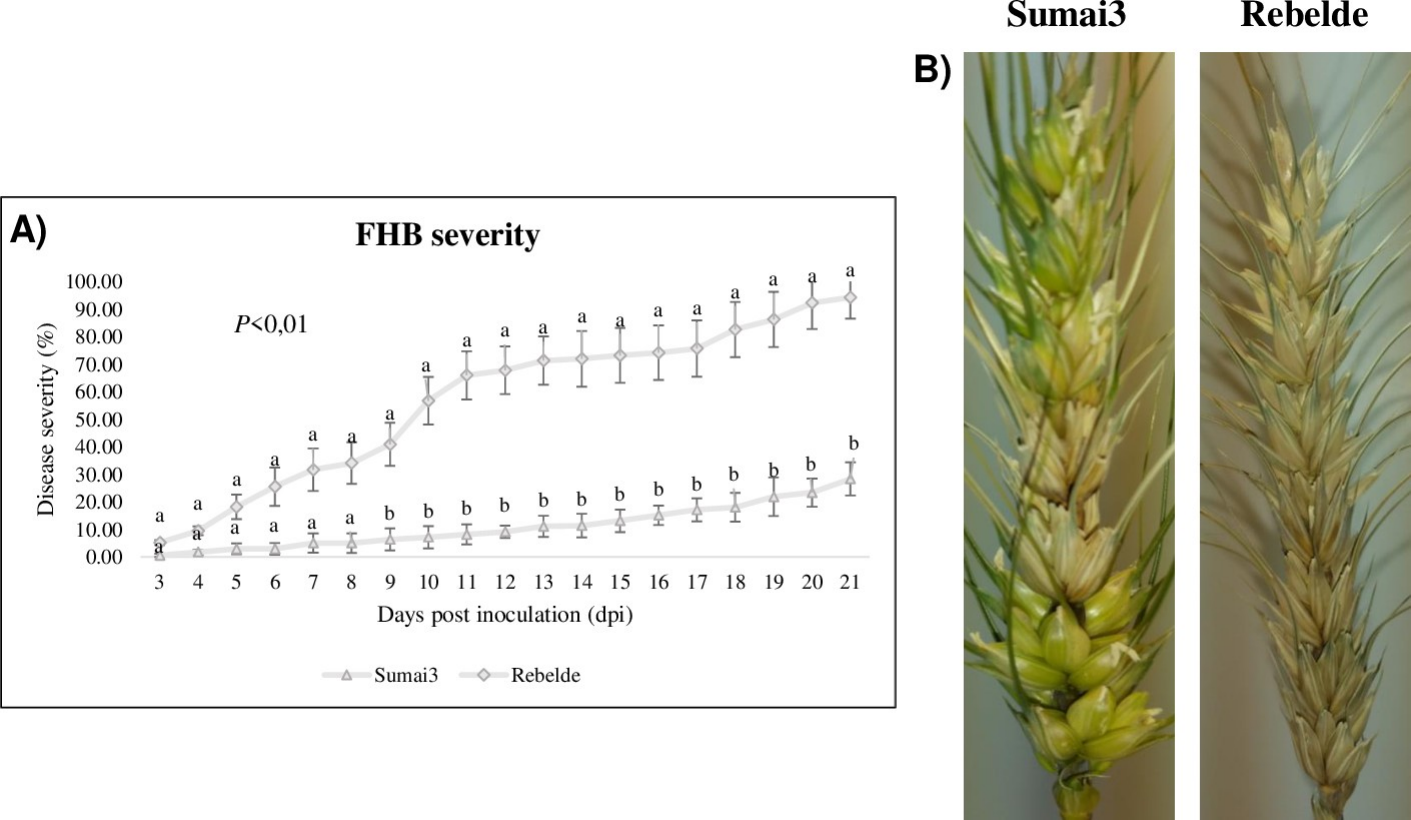
A) Fusarium head blight (FHB) severity (%) in Sumai3 and Rebelde from 3 to 21 days post inoculation (dpi). B) FHB symptoms in Sumai3 and Rebelde at 21 dpi. The data represent averages and standard errors from three independent replicates with at least 20 plants for each genotype. Different letters refer to the statistical analysis performed using one-way analysis of variance (ANOVA) with the Tukey test at a confidence level of 0.99 and *P*<0.01.

FHB severity (%) is represented in Fig 1A. From 3 to 8 dpi no statistical differences between the two wheat genotypes were recorded. From 9 to 21 dpi, FHB severity differed significantly, since at 21 dpi it reached 28% and 96% in Sumai3 and Rebelde, respectively. Fig 1B shows the symptoms at 21 dpi, while the bleaching remained confined to the central florets in Sumai3 but not in the Rebelde variety. No FHB symptoms were observed on the mock plants.

### Annotation, homology and phylogeny *in-silico* analyses of stomatal regulation genes

In the present study we isolated and annotated the genes involved in stomatal regulation from the bread wheat Rebelde and Sumai3 and we performed a homology and phylogeny analysis with related species. The amplification results shown that all the selected genes were successfully isolated from Rebelde and Sumai3 (S1 Fig). The sequencing results confirmed the unicity of the sequences from both genotypes. S2 Fig shows the homology and phylogeny trees of the genes selected. The closely-related sequences from Chinese Spring, *A*. *tauschii*, barley, Rebelde and Sumai3 mostly clustered together, while sequences from rice and maize represented the second major cluster. In particular, the genes of interest from Sumai3 and Rebelde showed a homology ranging from 87% to 100%. S3 Fig represents the alignment between Sumai3 and Rebelde of the genes having a homology equal or less than 95%. The major differences in the nucleotide sequence resides in SNPs mostly localized in intronic regions. A similar homology rate (87–100%) was also reported with Chinese Spring, *A*. *tauschii* and *H*. *vulgare*. *O*. *sativa* and *Z*. *mays* resulted to be the most distance species, since the homology rate with Rebelde and Sumai3 was of 69–86%.

### Establishment and validation of RT-*q*PCR

S4 Fig shows RNA integrity and *TaACT* amplifications from Rebelde and Sumai3, demonstrating that cDNA was successfully synthetized. The E and R^2^ values (S3 Table) for each primer pair were calculated: E ranged between 0.9056 to 1.1826 and 0.9759 to1.2463, R^2^ between 0.9907 to 0.9984 and 0.9891 to 0.9969 in the case of the amplification reactions obtained from Rebelde and Sumai3, respectively. E and R^2^ were not calculated for *TaTUB* from Rebelde because Cqs were detected only for 1:1 (36.65) and 1:10 (39.51) serial dilution (S4 Table) and the amplification obtained from the 1:10 dilution was considered negative (Cq > 38). The E and R^2^ values for *TaACT* were 1.1748 and 0.9954 for Rebelde and 1.1694 and 0.9961 for Sumai3, while E and R^2^ for *TaFNR* were 0.8988 and 0.9835 for Rebelde and 0.9249 and 0.9661 for Sumai3 (S3 Table). Standard errors (SE) among the Cq values for *TaACT* were ±0.194 to ±0.266 and ±0.311 to ±0.357 for Rebelde and Sumai3, respectively, while for *TaFNR* was ±2.014 to ±2.962 and ±2.122 to ±2.665. Hence, *TaTUB* and *TaFNR* were not taken into account for normalization (S4 Table).

### Differential expression patterns of the genes involved in stomatal regulation

**Fig 2:**
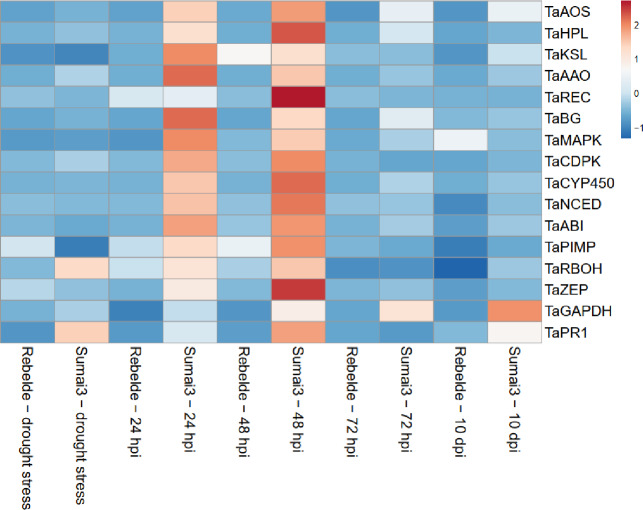
Heatmap related to the relative expression level of the genes selected. The relative expression levels are referred to the different plant treatments (drought stress, 24, 48, 72 hours post inoculation and 10 days post inoculation) and to the two wheat genotypes Sumai3 and Rebelde. The heatmap was constructed by analysing data with ClustVis software.

Fig 2 shows the heatmap for the relative expression values for each gene, genotype and type of stress applied to the plants, while S5 Table provides the relative expression values, SE and statistical analysis according to the Tukey test computed at 0.99 confidence level. Drought stress did not induce the selected genes, with the exception of *TaZEP* (2.638 and 1.533-fold in Rebelde and Sumai3), *TaBG* (4.313-fold) and *TaCYP450* (1.743-fold) in Sumai3. *TaPR1* was basally regulated in Rebelde (0.816-fold) but strongly up-regulated in Sumai3 (55.270-fold).

**Fig 3:**
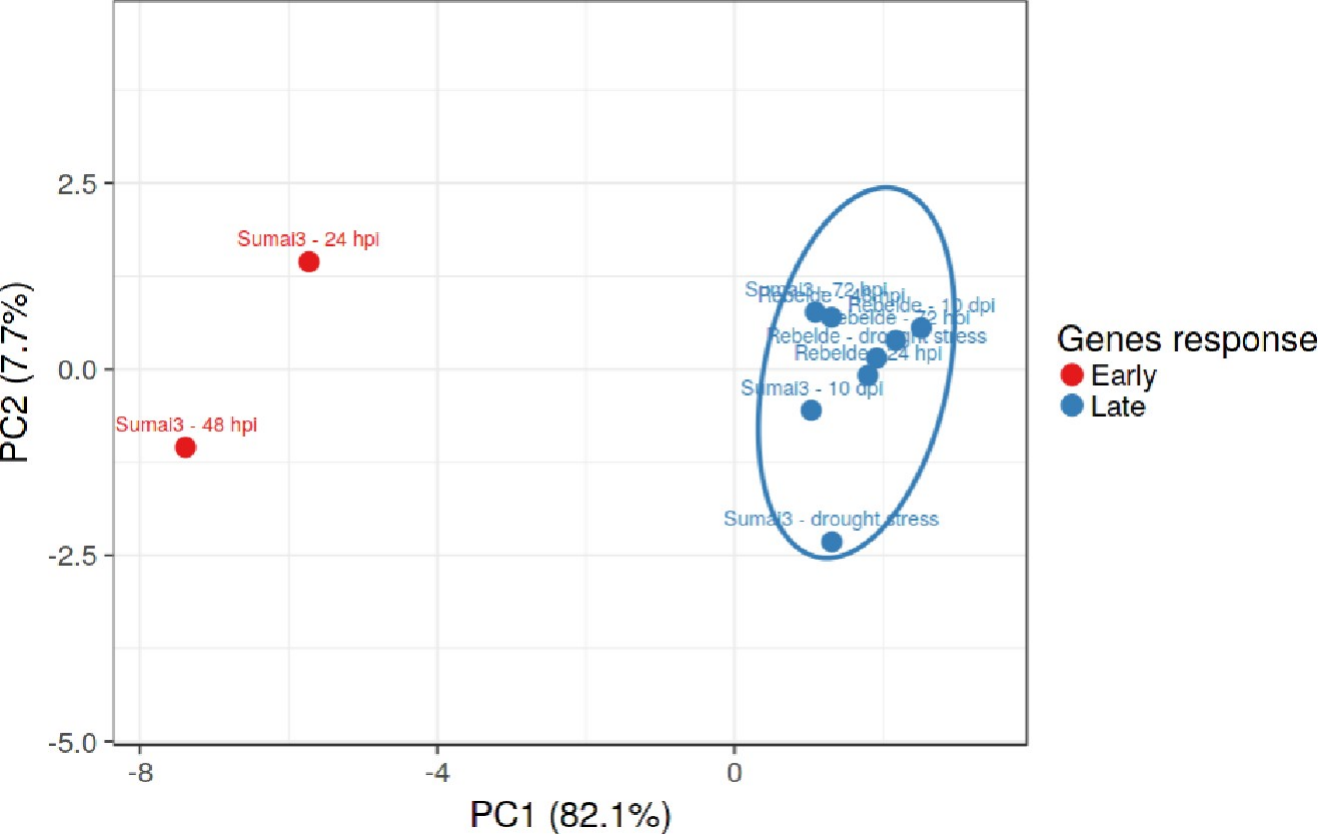
Principal Component Analysis (PCA) of the different plant treatments (drought stress, 24, 48, 72 hours post inoculation and 10 days post inoculation) and wheat genotypes (Sumai3 and Rebelde). The principal components 1 and 2 explain the 82.1% and 7.7% variability levels. PCA was conducted by using ClustVis software.

Between terminal drought stress and *F*. *graminearum* inoculation, the regulation of the selected genes was not extremely dissimilar within Rebelde, with the exception of *TaHPL*, which was strongly down-regulated at 10 dpi (0.009-fold), *TaKSL* slightly up-regulated at 48 hpi (1.617-fold), *TaREC* basally regulated at 24 hpi (1.048-fold) while down-regulated under drought stress (0.381-fold), *TaBG* up-regulated at 10 dpi (5.668-fold), *TaMAPK* basally regulated at 48 hpi (1.412-fold) and 72 hpi (1.098-fold) and up-regulated at 10 dpi (3.742-fold), *TaCYP450* progressively down-regulated from 48 hpi to 10 dpi (0.657–0.029-fold), *TaZEP* strongly down-regulated at 10 dpi (0.035-fold). Conversely, the expression pattern found among Sumai3 differed particularly when comparing drought stress and the different time of inoculation. Most of the selected genes were up-regulated at 24 and 48 hpi while *TaBG* and *TaCYP450* were considerably up-regulated (70.644 and 50.534-fold at 24 hpi and 39.768 and 53.172-fold at 48 hpi, respectively). At 72 hpi and 10 dpi some genes were down-regulated with the exception of *TaHPL*, *TaZEP*, *TaAOS*, *TaBG*, *TaMAPK* and *TaCYP450*. As a result, the difference between the two genotypes was obvious, since the genes involved in stomatal regulation promptly responded to *F*. *graminearum* in Sumai3 but not in Rebelde, suggesting their involvement in FHB resistance. Furthermore, *TaHPL*, *TaKSL*, *TaAAO*, *TaREC*, *TaCDPK*, *TaNCED*, *TaABI*, *TaPIMP* and *TaZEP* seemed to be involved in early response, while *TaAOS*, *TaBG*, *TaMAPK* and *TaCYP450* in long-term response to *F*. *graminearum*. Additionally, principal-component analysis (PCA) was carried out to gain in-depth understanding of the role of the genes selected and Fig 3 shows that two main clusters have been identified as deriving from the PCA. Relative expression values for Sumai3 at 24 and 48 hpi clustered together, confirming the hypothesis that most of the genes are involved in an early response to *F*. *graminearum*. *TaPR1* was progressively up-regulated in both the genotypes, as expected. *TaGAPDH* was basal regulated under drought-stress condition in both the wheat genotypes and under *F*. *graminearum* infection in Rebelde, while progressively up-regulated in Sumai3 at 24, 48, 72 hpi and 10 dpi.

### Phenotypical parameters of stomatal regulation

**Fig 4:**
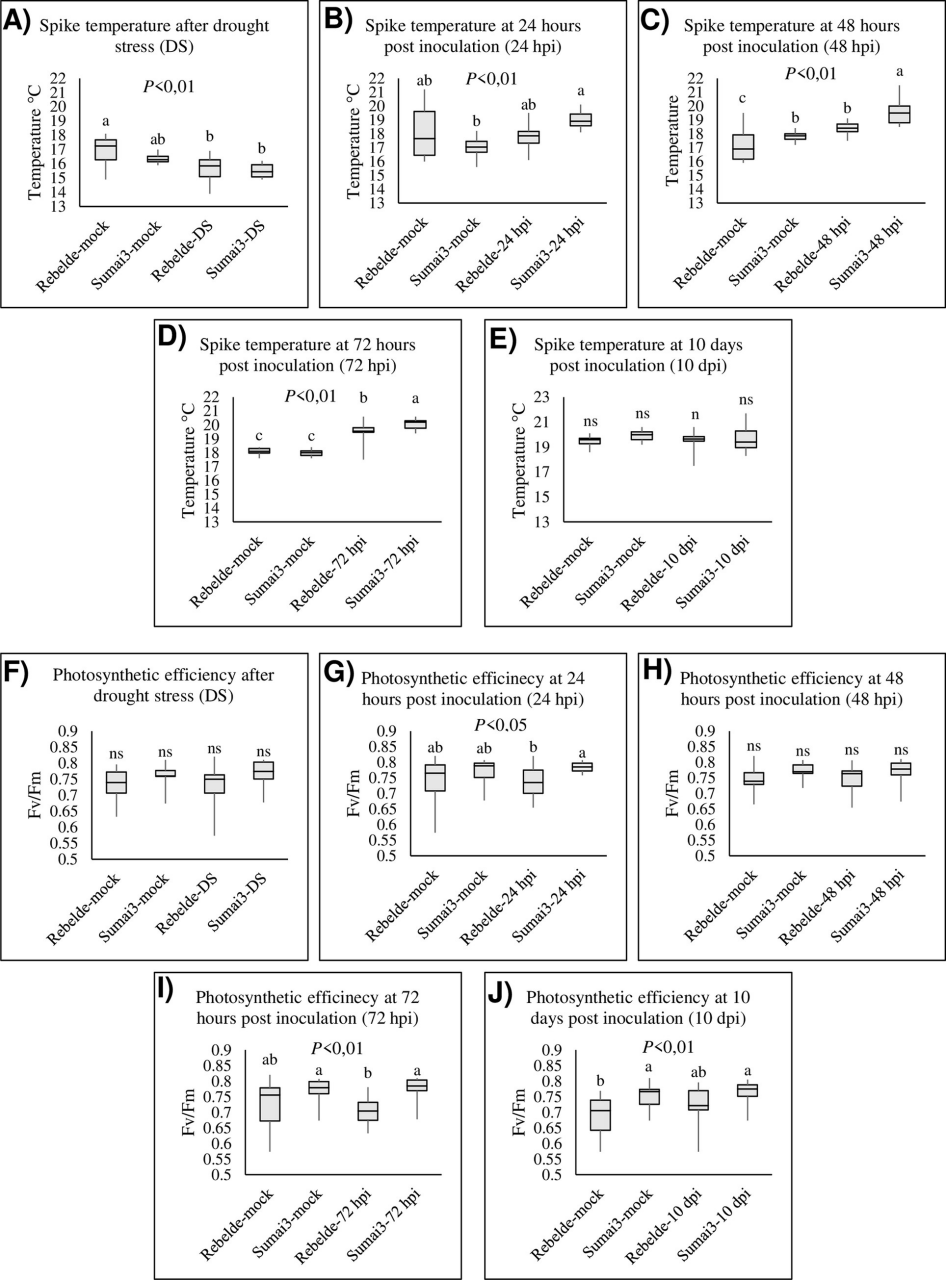
Box-plots of photosynthetic phenotypic parameters measured for each wheat genotype (Sumai3 and Rebelde) and each different plant treatment. A-E represent spike temperatures (°C) from plants subjected to drought stress (A), 24 hours post inoculation (B), 48 hours post inoculation (C), 72 hours post inoculation (D) and 10 days post inoculation (E). F-J represent the photosynthetic efficiency (Fv/Fm) of plants subjected to drought stress (F), 24 hours post inoculation (G), 48 hours post inoculation (H), 72 hours post inoculation (I) and 10 days post inoculation (J). Fv/Fm represents the variable-to-maximum-fluorescence ratio. The data represent averages and standard errors for three independent replicates with at least 20 plants for each genotype. Different letters refer to the statistical analysis performed using one-way analysis of variance (ANOVA) with the Tukey test at 0.95 or 0.99 confidence level and *P*<0.05 or *P*<0.01, while “ns” refers to not significant differences.

**Fig 5:**
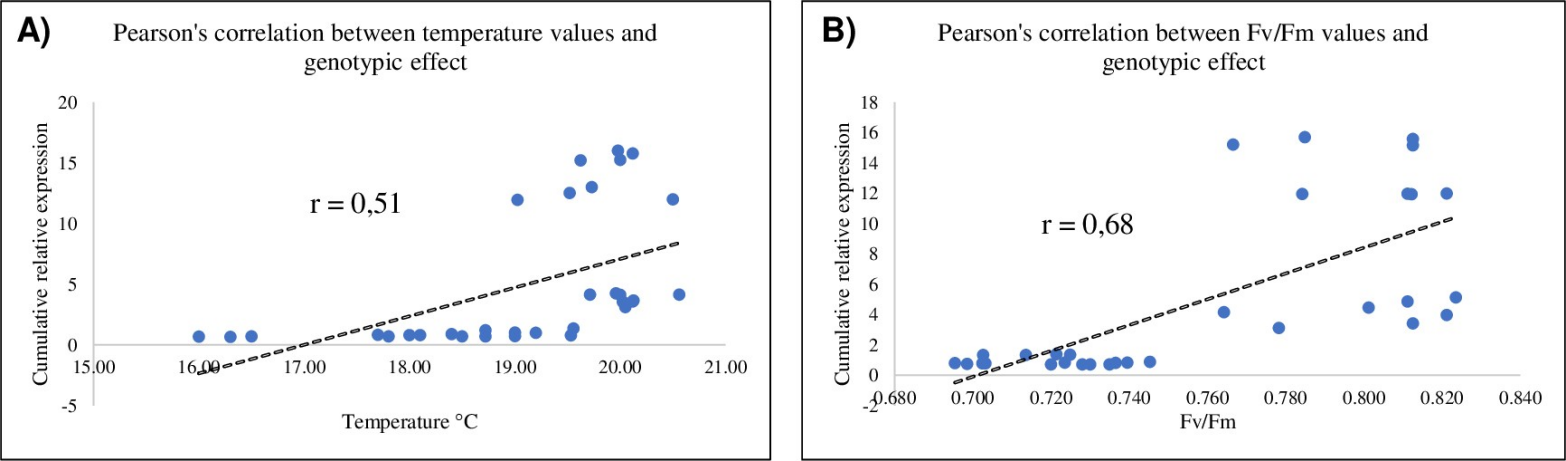
Pearson’s correlation between A) temperature values and genotype; B) Photosynthetic efficiency (Fv/Fm) and genotype. Data show that higher temperature and photosynthetic efficiency values are positively correlated with an increasing of transcript levels in Sumai3 under *F*. *graminearum* inoculation. The Pearson’s correlation was computed for *P<*0.05.

Fig 4A–4E show the spike temperature reached after the plants were subjected to drought (A), 24 hpi (B), 48 hpi (C), 72 hpi (D) and 10 dpi (E). Spike temperatures after drought stress did not increase (15.60°C and 15.49°C, while the mock plants reached 16.88°C and 16.34°C respectively in the Rebelde and Sumai3). Spike temperatures increased gradually from 24 hpi to 72 hpi in Sumai3 (19.03°C, 19.63°C and 20.05°C at 24, 48 and 72 hpi, while the mock plants reached 17.01°C, 17.79°C and 17.98°C). The temperature increased significantly also in Rebelde at 48 (18.40°C, while the mock plants reached 17.27°C) and 72 hpi (19.53°C, while the mock plants reached 18.09°C), suggesting that the few slightly up-regulated and basally regulated genes in the susceptible wheat genotype may contribute to stomatal closure. Fig 4F–4J show the photosynthetic efficiency of the spikes after the plants were subjected to drought (F), 24 hpi (G), 48 hpi (H), 72 hpi (I) and 10 dpi (J). Their photosynthetic efficiency was not significantly affected by drought and did not vary at 48 hpi, while the two genotypes differed significantly among themselves at 24 and 72 hpi but not compared to their respective mock controls. Sumai3 maintained a higher photosynthetic efficiency than Rebelde at 24 (0.784 and 0.735, respectively) and 48 hpi (0.778 and 0.702, respectively). Moreover, the observations performed by using the optical microscopy demonstrated that stomata remained open under mock and drought conditions and at 10 dpi in both the wheat genotypes, they were partly open at 24, 48 and 72 hpi in Rebelde and closed at the same time-points in Sumai3 (S5 Fig). Additionally, the Pearson’s correlation (Fig 5A and 5B) revealed that there is a moderate positive correlation between the temperature variation and the genotypic effect (Fig 5A) and a strong positive correlation between the photosynthetic efficiency variation and the genotypic effect (Fig 5B) under the inoculation condition. These results confirmed that the increasing of temperature and photosynthetic efficiency are closely related to resistance to FHB.

## Discussion

### Annotated functions of the genes involved in stomatal regulation

In the present study we isolated and annotated the genes involved in stomatal regulation from the bread wheat Rebelde and Sumai3 and we performed a homology and phylogeny analysis with related species. TPS is the enzyme involved in terpenoid biosynthesis, the precursors of ABA. TPS enzymes were largely studied in rice, where it is well known that they accumulate strongly in plant tissues after mechanical damage, but very little is known in other cereal-related species. In this study, a *TPS* sequence annotated as *ent*-kaurene synthase *KSL* was identified [38]. The next reaction modulating stomatal movements is catalysed by ZEP. *ZEP* was the first gene isolated from Arabidopsis among the genes involved in stomatal regulation and was up-regulated during water stress [39]. The signalling pathway continues with the activation of *NCED*. NCED has been extensively characterized in Arabidopsis. In bread wheat, two genes have been cloned, *TaNCED1* and *TaNCED2*, responding to low temperature, drought, NaCl and ABA application [40]. AAOs catalyses the final step of carotenoid catabolism. AAOs are involved in the last reaction for positively regulating the stomatal closure. In wheat, three *AAO* isoforms were annotated, showing a central role in the accumulation of carotenoids in kernels [41]. The CYP450 superfamily is the largest enzymatic protein family found in plants and is involved in the biosynthesis of diverse plant pigments, such as carotenoids and anthocyanins, as well as the regulation of hormone metabolism [42]. In bread wheat, two genes annotated as *TaCYP78A3* and *TaCYP707A1B* have been characterized; the functionality of *TaCYP78A3* is strongly correlated with kernel size, while *TaCYP707A1B* affected ABA level and dormancy in seed [43]. BG plays a key role both as positive and negative regulator of stomatal closure by conjugating and deconjugating ABA. BG was up-regulated by drought stress and its knockdown caused defective stomatal movement, early germination and abiotic stress-sensitive phenotypes [44]. BG has been also widely investigated for its antifungal activity, since it has been classified as a pathogenetic-related protein [45]. Among the large family of plant receptors, the *REC* family was amply characterized in Arabidopsis and rice as a class of membrane-localized receptors [46,47]. In wheat, two *REC*s were identified and one of them, *Ta_PYL4AS_A*, seemed to be associated with FHB susceptibility [48]. RECs are able to interact with ABI to modulate the stomatal movements. ABIs are protein phosphatase type 2C class (PP2Cs) and have been investigated for their functions regarding plant growth, development and stress responses in Arabidopsis [46]. The resulting signalling pathway generated from the different modulation of the previous genes activates the cascade of CDPKs and MAPKs. The CDPKs represent a class of Ca^2+^ sensors capable of modulating its intracellular levels in response to hormones, light, abiotic stress and pathogen elicitors [49]. In wheat, 20 *CDPK*s have been identified, responding to cold, hydrogen peroxide, ABA, gibberellic acid (GA), powdery mildew and sharp eyespot [50]. MAPK pathways are relevant actors in signal transduction events, functioning downstream as sensors/receptors and converting signals into cellular responses [51]. In bread wheat, 54 *MAPK*s have been identified and 84% of these were up-regulated by heat, cold, drought or salt stress [52]. Activation of MAPKs as modulator of stomatal movements is linked to the production of ROS, leading to an establishment of a positive feedback loop involving the up-regulation of ROS-producing and antioxidant genes [53]. ROS have been identified as key molecules positively regulating stomatal closure and several studies in Arabidopsis, maize and wheat have identified RBOHs as a source of ROS in guard cells. The roles of ROS in plant defence have been studied in detail, since exposure of plant cells to avirulent pathogens or elicitors triggers a rapid and prolonged oxidative burst [54]. In bread wheat, 46 *RBOHs* were identified as participants in cell-wall biosynthesis, defence responses and signal transduction [55]. JA and MeJA play a key role in stomatal regulation. *AOS*, *HPL* and *MYB* represent the core of the JA cascade. In bread wheat, three copies of *AOS* have been cloned [56]. AOS is strictly related to HPL activity [57]. *AOS* and *HPL* were first attributed to the same gene family, until *HPL* was characterized in Arabidopsis, rice and maize as a protein strongly accumulated in the inflorescence and localized in plastids [58]. MYB transcription factors are regulatory proteins implicated in plant growth, secondary metabolism, hormone signal transduction, disease and abiotic stress resistance were identified [59,60]. In bread wheat, 23 gene fragments and 6 nearly complete open-reading frames (ORFs) encoding putative *MYBs*, implicated in providing to tolerance to salt, in lignin biosynthesis, fructan accumulation and increased spike and grain weight were isolated. In the present study, we identified a *MYB* sequence previously annotated as *TaPIMP* [61].

### Expression responses to drought stress and *F*. *graminearum* inoculation

Transcriptional analysis revealed that drought stress did not induce the selected genes. Although we hypothesised that the stomatal closure promoting genes were up-regulated because of drought stress, many studies are in agreement with our results which support the hypothesis that hydric conditions induce stomatal closure in drought-sensitive wheat varieties. By way of contrast, drought-tolerant cultivars correlate with lower level of closure-inducing genes and higher expressions of genes negatively regulators of stomatal closure [62]. Therefore, since we observed a basal regulation of *TaBG*, *TaCYP450* and *TaGAPDH*, while only *TaZEP* was up-regulated in Rebelde, and a slight up-regulation of the same genes in Sumai3, we can hypothesise that the two wheat genotypes could be drought-tolerant, especially due to the fact that GAPDH is particularly sensitive to changes in photosynthesis by abiotic and biotic stresses [35,63]. These results could be supported by three studies, whereas in drought-tolerant wheat cultivars the up-regulation of *CYP* and *BG* and the down-regulation of *ZEP*, *NCED* and *AAO* have been demonstrated [64–66].

Many transcriptomic research studies have embraced the concept of comparing the response of a resistant and susceptible genotype to *Fusarium* spp. infection, in order to explore and uncover the plant-pathogen molecular responses. Most of the transcriptomic studies pointed out that the establishment of an early and prompt defence response is a decisive feature that contributes to FHB resistance [67–69]. Soresi et al. (2015) found that several stomatal movements responsive elements were induced in the resistant Langdon wheat line but not in its susceptible parental line [70]. Dhokane et al. (2016) established a transcriptome analysis revealing that MYB transcription factors were up-regulated only in the resistant wheat genotype carrying the Fhb2 [71].

Much evidence supported our results indicating that the *CYP450* gene family plays an active role in wheat resistance against *F*. *graminearum* and DON. In actual fact, strong *CYP450* accumulations were found in resistant but not in susceptible wheat cultivars challenged with *F*. *graminearum* and DON [72,73]. CYP450s are involved in the detoxification of xenobiotic substances. Hence, the induction of *CYP450* by *F*. *graminearum* in the resistant wheat genotype might denote a resistant physiological mechanism to FHB toxic metabolites, such as the mycotoxin DON. This hypothesis was confirmed by several studies demonstrating that CYP450s were able to detoxify DON *in vitro* [74] and *in vivo* [73]. De Zutter et al. (2017) investigated plant-response in wheat ears to a combined attack of *F*. *graminearum* and aphids and observed that *PR1* and *PR2*, the latter encoding a β-1,3-glucanase, were consistently up-regulated [75]. Our results are in agreement with those obtained by the previously cited authors. Of the negative regulator genes, *TaREC* was mainly down-regulated. This may be due to the possibility that *TaREC* may be involved in FHB susceptibility. Gordon et al. [48] found that *REC* silencing in bread wheat resulted in lower FHB symptoms progression and decreased DON content in wheat heads [48]. JA and its derivate MeJA, are positive regulators of FHB resistance in wheat and CDPKs are the enzymes favouring ABA and JA crosstalk. There is some evidence that *CDPK*s may be involved in wheat responses to some fungal diseases, like powdery mildew and sharp eyespot, but further information regarding FHB is not available [50,76]. We also investigated the role of *TaMAPK*, which was induced in Sumai3 at 24 and 48 hpi. In wheat, a gene labelled as *TaFLRS*, turned out to be a MAP kinase transcriptionally up-regulated in *F*. *graminearum* inoculated spikes [77]. As far as we know, no information regarding the role or the mode of action of *TaKSL*, *TaZEP*, *TaNCED*, *TaAAO*, *TaABI and TaHPL* as responsive genes during wheat-*F*. *graminearum* interaction is available. Therefore, this work provided evidence for the first time that these genes are induced by *F*. *graminearum* in Sumai3, suggesting its role in FHB resistance.

Our research seems to make clear that the induction of stomatal regulation genes is a characteristic of the resistant Sumai3 genotype. The two QTLs most effective in contrasting FHB, *Fhb1* and *Qfhs*.*ifa-5A*, are derived from the genome of Sumai3, playing a key role in the regulation of FHB-responsive genes. Both QTLs contribute to FHB resistance by inducing several genes including *MYC*, *MYB* transcription factors and *AOS* [20,78,79].

Despite the fact that the function of ABA as a response to abiotic stress has been well documented, its role in plant defence is more obscure. For instance, ABA seems to have an ambivalent role during the pathogen defence mechanisms; hence, its role as positive or negative regulator during the pathogen infection has not already been disclosed [80]. Nevertheless, the bulk of evidence is leaning more toward ABA as a susceptible factor, at least with respect to fungal pathogens [80–82]. The role of ABA and *TaREC* in mediating susceptibility to FHB might explain why the genes involved in the negative regulation of stomatal closure (*TaCYP450* and *TaBG*) were importantly up-regulated in Sumai3, while *TaREC* was mainly down-regulated in the resistant wheat line.

### Phenotypical responses to drought stress and *F*. *graminearum* inoculation

After drought stress, neither spike temperature nor photosynthetic efficiency were negatively affected, reflecting the results obtained by transcript analysis, supporting the hypothesis that the two genotypes tolerate hydric stress. A morphological explanation for wheat tolerance to drought was provided by Ding et al. (2017), who observed that stomata were equally present on the abaxial and adaxial sides of glumes and lemmas, providing the first evidence of amphistomatous traits of wheat ears, which might explain how wheat spikes can maintain high photosynthetic rates even under conditions of drought [83]. Several plant metabolic pathways like photosynthesis change as an infection is established [84–86]. Hence, chlorophyll fluorescence has been already used with success to estimate the fungal and bacterial disease severity, suggesting that changes in the chlorophyll metabolism and photosynthesis-related parameters are associated to the infection establishment [87,88]. However, to date, the specific connection between *F*. *graminearum*-wheat interaction and photosynthetic parameters has not been sufficiently demonstrated. One consistent research study has evaluated the effect of *F*. *graminearum* infection on the photosynthetic rate of wheat flag leaves in resistant and susceptible lines. The authors observed that FHB caused more significant reduction in the net photosynthetic rate of flag leaves in the resistant line [23]. These results are not in agreement with those observed by us in the present study, but this discordance may be due to several factors affecting the experimental settings. In actual fact, the authors in question evaluated the parameters at 7 and 21 dpi on the flag leaves while we considered earlier time-points in spikes. Hence, the present work represents the first evidence that connects the increasing spike temperatures with putative FHB resistance. Therefore, we might hypothesize that increases in spike temperatures at 24, 48 and 72 hpi and in photosynthetic efficiency at 24 and 72 hpi in Sumai3 compared to Rebelde, may be linked to a differential rate of stomatal conductance regulation mediated by an early FHB resistance response. This physiological mechanism could lead to an enhanced photosynthesis related to FHB resistance, as supported by the induction of transcript levels of *TaGAPDH* in the present study and as already observed by Zhang et al. (2013), who reported the accumulation of the protein after 72 hpi [63].

## Conclusions

In the present work we firstly reported that most of the genes involved in the positive regulation of stomatal closure were induced in the resistant cultivar Sumai3 after *F*. *graminearum* inoculation. Nevertheless, *TaBG* and *TaCYP450*, negative regulators of stomatal closure, were even stronger up-regulated. Thus, we conclude that stomatal closure is involved in resistance to FHB in wheat by an intense cross-talk among positive and negative genes regulating the guard cells movements. The transcriptional changes also resulted in modification of photosynthetic parameters resulting in an increasing of spikes temperature, as an outcome of stomatal closure, in both of the wheat genotypes and in a decreasing of photosynthetic efficiency in Rebelde but not in Sumai3, confirming that photosynthetic parameters are related to FHB resistance.

## Supporting information

S1 Table A list of selected genes, genomic location, accession numbers, gene functions, primer pairs and amplicon lengths from DNA and cDNA. Primer pairs used in Real-Time *q*PCR are identified by the letter “q” in their names, with the exception of primer pairs for *TaGAPDH*, *TaPR1*, *TaACT*, *TaTUB* and *TaFNR*, where the same primer pairs were used both for PCR and Real-Time *q*PCR. The genomic location derived from the IWGSC database (IWGSC_ref_v1) (https://wheat-urgi.versailles.inra.fr/). while the accession numbers refer to the queries that matched on BLASTn (https://blast.ncbi.nlm.nih.gov/).
(DOCX)

S2 Table Genomic location of homologous sequences used to obtain homology and phylogeny trees. The full-length sequences were extracted from Gramene (www.gramene.org).
(DOCX)

S3 TableAmplification Efficiency (E) and R^2^ values for each selected gene amplified taken from each wheat genotype (Rebelde and Sumai3).The E and R^2^ values were calculated as follows: five 1:10 serial dilutions (1:1–1:10000) were obtained for each sample of cDNA and amplified in four technical replicates obtained from four independent biological replicates. E and R^2^ values were calculated by the slope of the standard curve obtained by plotting fluorescence versus serial dilution concentrations using the equation $E=10^{\left(-\frac{1}{slope}\right)}-1$. The E and R^2^ values for the *TaTUB* taken from Rebelde were not calculated (x), because amplification did not occur at 1:100, 1:1000 and 1:10000 cDNA dilutions.(DOCX)

S4 Table Quantification Cycles (Cq) and relative Standard Errors (SE) obtained from five 1:10 serial dilutions of cDNA and used to evaluate the stability of each reference gene (*TaACT*, *TaTUB* and *TaFNR*) chosen from among the different plant treatments (mock, drought stress, 24 hpi, 48 hpi, 72 hpi and 10 dpi). Cq values were calculated from four technical replicates derived from four independent biological replicates. *TaTUB* from Rebelde did not occur at 1:100, 1:1000 and 1:10000 cDNA dilutions, so no Cq values were detected (nd), therefore, the SE was not calculated (x).
(DOCX)

S5 Table Relative expression levels and standard errors of each gene selected for each genotype (Rebelde and Sumai3) and for each plant-treatment mode (drought stress, 24 hpi, 48 hpi, 72 hpi and 10 dpi). Relative expression values were obtained by using the equation 2^-ΔΔCq^ with *TaACT* as a reference gene and mock treatments used to normalize the relative expression levels. The data represent averages and standard errors for the four independent biological replicates and the four technical replicates examined. The data were subjected to two-way analysis of variance (ANOVA), where the two independent variables were the wheat genotype (Rebelde and Sumai3) and the plant treatment (drought stress, 24 hpi, 48 hpi, 72 hpi and 10 dpi) while the dependent variable was the relative expression level. Different letters among the plant treatments and wheat genotypes were assessed by means of significant F values by applying the Tukey Honestly Significant Difference test (Tukey test) at 0.99 confidence level (*P*<0.01).
(DOCX)

S1 Fig 1.5% agarose gel of selected genes amplified by using primer pairs for Real-Time *q*PCR reported in S1 Table. M) ExcelBand™ 100 bp + 3K DNA Ladder (Smobio), 1) *TaAOS*, 2) *TaABI*, 3) *TaACT*, 4) *TaPR1*, 5) *TaKSL*, 6) *TaBG*, 7) *TaPIMP*, 8) *TaREC*, 9) *TaCYP450*, 10) *TaZEP*, 11) *TaHPL*, 12) *TaCDPK*, 13) *TaNCED*, 14) *TaRBOH*, 15) *TaAAO*, 16) *TaMAPK*, 17) *TaTUB* from Rebelde (not detected), 18) *TaTUB* from Sumai3, 19) *TaFNR* from Rebelde, 20) *TaFNR* from Sumai3, 21) *TaGAPDH* from Rebelde. The Fig represents the original picture of the gel.
(DOCX)

S2 Fig Homology and phylogeny trees of the selected genes. Homology trees are represented in Figs from A-I to N-I and phylogeny trees are represented in Figs from A-II to N-II. The trees were obtained by clustering nucleotide sequences isolated using the primer pairs listed in S1 Table. The trees were constructed using DNAMAN software (Lynnon Biosoft, Quebec, Canada).
(DOCX)

S3 Fig Alignment of sequences from Rebelde and Sumai3. The sequences with a homology equal or less than 95% (*TaKSL*, *TaZEP*, *TaNCED*, *TaAAO*, *TaAOS* and *TaPIMP*) were aligned in order to observe the major pieces of the sequences characterized by SNPs.
(DOCX)

S4 Fig 1.5% agarose gel of total extracted RNA (A, C) and RT-PCR (B, D) from Rebelde and Sumai3, respectively. M) ExcelBand™ 100 bp + 3K DNA Ladder (Smobio), 1) Mock, 2) Drought stress, 3) 24 hpi, 4) 48 hpi, 5) 72 hpi, 6) 10 dpi, 7) Genomic DNA (gDNA) control, 8) No template control (NTC). The Fig represents the original picture of the gel.
(DOCX)

S5 Fig Stomata from Sumai3 and Rebelde. The pictures were obtained by observing a slice of the external glume with an optical microscope (Leitz Diaplan) and by using a magnification of 40X.
(DOCX)

S1 Raw materials (PDF)
