# Supplementary material for: The modulation of stomatal conductance and photosynthetic parameters is involved in Fusarium head blight resistance in wheat
Source: PLoS One. 2020 Jun 30;15(6):e0235482. doi: 10.1371/journal.pone.0235482 (PMC7326183; doi:10.1371/journal.pone.0235482)
Supplement: S1 Table — Primer pairs used in Real-Time qPCR are identified by the letter “q” in their names, with the exception of primer pairs for TaGAPDH, TaPR1, TaACT, TaTUB and TaFNR, where the same primer pairs were used both for PCR and Real-Time qPCR. The genomic location derived from the IWGSC database (IWGSC_ref_v1) (https://wheat-urgi.versailles.inra.fr/). while the accession numbers refer to the queries that matched on BLASTn (https://blast.ncbi.nlm.nih.gov/). (DOCX) [file pone.0235482.s001.docx]

**S1 Table**

| **Gene, genomic location and accession number** | **Function** | **Primer name** | **Primer pairs (5'-3')** | **bp (DNA)** | **bp (cDNA)** |
| --- | --- | --- | --- | --- | --- |
| *TaAOS*  4B:495147007-495149759  BLASTn: AY196004 | Allene oxide synthase | TaAOS_F | GTTAGCTGCTCGTGATCGAT | 1270 |  |
|  |  | TaAOS_R | TGCAGCAGCTTGCTTCTCTC |  |  |
|  |  | TaAOS_qF | TCGGGCGTATTGCTGAGG | 352 | 352 |
|  |  | TaAOS_qR | TGCAGCAGCTTGCTTCTCTC |  |  |
| *TaHPL*  6D:13059842-13062314  BLASTn: AK335301 | Hydroperoxide lyase | TaHPL_F | GCAGCTGGCCCAGTGAGCT | 1520 |  |
|  |  | TaHPL_R | GTGAAGGCGGTGCCGTCGCA |  |  |
|  |  | TaHPL_qF | GGACCACAGCATGACCGACA | 190 | 190 |
|  |  | TaHPL_qR | GAACCCGACCTCGCCGTT |  |  |
| *TaKSL*  Un:12441012-12441346  BLASTn: AB597957 | Terpene synthase | TaKSL_F | GGAACGGGATGCTAGAATACG | 2075 |  |
|  |  | TaKSL_R | TCTCTAACAACCATCCTTAG |  |  |
|  |  | TaKSL_qF | GGAACGGGATGCTAGAATACG | 282 | 282 |
|  |  | TaKSL_qR | CCTCTCCTTATGTGGTCGGA |  |  |
| *TaAAO*  7B:687590126-687591793  BLASTn: AK331622 | ABA-aldehyde oxidase | TaAAO_F | TCGACGTTGCACAACACGAT | 2575 |  |
|  |  | TaAAO_R | GTAGTCTACAGTTCATCACA |  |  |
|  |  | TaAAO_qF | CCTGAGCAATCAAAGCATCCC | 182 | 182 |
|  |  | TaAAO_qR | TCAGGCTGACACGCTGAACT |  |  |
| *TaREC*  2D:37645390-37646533  BLASTn: AK335719 | ABA receptor | TaREC_F | GCTCCGACCTCGAGCCGATG | 1020 |  |
|  |  | TaREC_R | ATCTTATCATCATCTTAC |  |  |
|  |  | TaREC_qF | GCTGGAGATCCTGGACGAC | 217 | 217 |
|  |  | TaREC_qR | GTTGCACTTGACGATGGTGT |  |  |
| *TaBG*  3B:771171542-771172472  BLASTn: Y18212 | β-1,3-glucanase | TaBG_F | GCGTGATCGGCAACAACCTC | 855 |  |
|  |  | TaBG_R | CGAAGCTCCTCTCCGTGGCG |  |  |
|  |  | TaBG_qF | AACGTGCGCCCCTACTACC | 398 | 398 |
|  |  | TaBG_qR | GCGTCGAACAGGCTCGTGTA |  |  |
| *TaMAPK*  4A:120605915-120606435  BALSTn: AF079318 | Mitogen activated protein kinase | TaMAPK_F | ATCATCAGCTCAGCCTCAGC | 2618 |  |
|  |  | TaMAPK_R | TACAGACAGTTTTATACCGA |  |  |
|  |  | TaMAPK_qF | CATCGACGTCTGGTCCGT | 154 | 154 |
|  |  | TaMAPK_qR | GTCCTCGTTCCGGATGAATC |  |  |
| *TaCDPK*  2D:161373811-161376953  BLASTn: KU516994 | Calcium dependent protein kinase | TaCDPK_F | GGGAGCCTCGTGTGTTGGCG | 1647 |  |
|  |  | TaCDPK_R | GTACTTGTTATCTGCAACGA |  |  |
|  |  | TaCDPK_qF | CTTCTTTGTGGTGTCCCTCC | 436 | 436 |
|  |  | TaCDPK_qR | GCTGTCAAACGCCTCCTT |  |  |
| *TaCYP450*  7D:258236253-258238309  BLASTn: XM_020311851 | Cytochrome P450 | TaCYP_F | GTATTGGTGGACGAGGAAGG | 1810 |  |
|  |  | TaCYP_R | ACTGTCACACTCACTTCCTG |  |  |
|  |  | TaCYP_qF | GTATTGGTGGACGAGGAAGG | 411 | 411 |
|  |  | TaCYP_qR | ATCTGGCGTGCGATCACT |  |  |
| *TaNCED*  5A:572021505-572023279  BLASTn: KX711891 | Epoxycarotenoid dioxygenase | TaNCED_F | CGGTGGAGAGGCAGGAGAA | 1534 |  |
|  |  | TaNCED_R | CCGTGGAAGCCGTACGGCAC |  |  |
|  |  | TaNCED_qF | CGGTGGAGAGGCAGGAGAA | 472 | 472 |
|  |  | TaNCED_qR | CGCGCGTAGAACAGAGCA |  |  |
| *TaABI*  3B:378687185-378688024  BLASTn: AB238930 | Phosphatase | TaABI_F | ATGGAGGACGTGGCCGTGGC | 2450 |  |
|  |  | TaABI_R | TGTGCAATGGTAGTATAG |  |  |
|  |  | TaABI_qF | TAGCAGATTACTGTCGGGATCGG | 283 | 283 |
|  |  | TaABI_qR | GAGCAGATGACAGCGACCA |  |  |
| *TaPIMP*  1B:615731797-615736596  BLASTn: KX683396 | MYB domain transcription factor | TaPIMP_F | TGCCTAGCTCGTGGGAGTAG | 1307 |  |
|  |  | TaPIMP_R | ACTAATGGTATCAATGTTCA |  |  |
|  |  | TaPIMP_qF | GTCACAGATCGCGTCGCAC | 325 | 325 |
|  |  | TaPIMP_qR | GCATCCGAACTGGCCGTACA |  |  |
| *TaRBOH*  3A:509419957-509424756  BLASTn: AY561153 | NADPH oxidase | TaRBOH_F | CTTCTCAATTACTTCAGCAC | 1010 |  |
|  |  | TaRBOH_R | TAGTTCCAGAGACAACATCG |  |  |
|  |  | TaRBOH_qF | TTGTTGGATTAGGAATTGGTGCT | 441 | 280 |
|  |  | TaRBOH_qR | TGATCCATGTCGGCAATCTC |  |  |
| *TaZEP*  2A:542649865-542654664  BLASTn: AF384103 | Zeaxanthin epoxidase | TaZEP_F | AAGGTCGTGTCACCTTGCTT | 2590 |  |
|  |  | TaZEP_R | GGCACAACAATGTACTGTACTAGG |  |  |
|  |  | TaZEP_qF | CTTGTCACCAGCCACTGTAC | 236 | 236 |
|  |  | TaZEP_qR | GGCACAACAATGTACTGTACTAGG |  |  |
| *TaGAPDH –* KR029493.1 | Glyceraldehyde-3-phosphate dehydrogenase | TaGAPDH_F  TaGAPDH_R | AGTTCATGCCATGACTGCAA  CCAGTGCTGCTTGGAATGATG | 105 | 105 |
| *TaPR1 -* AJ007348 | Pathogenesis related protein-1 | TaPR1_F | ACTACGACTACGGGTCCAACA | 145 | 145 |
|  |  | TaPR1_R | TCGTAGTTGCAGGTGATGAAG |  |  |
| *TaACT -* AB181991 | Actin | TaACT_77F | TCCTGTGTTGCTGACTGAGG | 350 | 236 |
|  |  | TaACT_312R | GGTCCAAACGAAGGATAGCA |  |  |
| *TaTUB -* TAU76745 | β-tubulin2 | TaTUB_F | CGAGGAGGGCGAGTACGA | 79 | 79 |
|  |  | TaTUB_R | AGCAAAGCACGACATGGACAT |  |  |
| *TaFNR -* AJ457980 | Ferredoxin - NADP(H)-oxidoreductase | TaFNR_F | CACCGGCCCAGTGATCTT | 259 | 69 |
|  |  | TaFNR_R | AAGGGCGTCTGCTCCAACT |  |  |
