# Supplementary material for: The modulation of stomatal conductance and photosynthetic parameters is involved in Fusarium head blight resistance in wheat
Source: PLoS One. 2020 Jun 30;15(6):e0235482. doi: 10.1371/journal.pone.0235482 (PMC7326183; doi:10.1371/journal.pone.0235482)
Supplement: S2 Table — The full-length sequences were extracted from Gramene (www.gramene.org). (DOCX) [file pone.0235482.s002.docx]

**S2 Table**

| **Species/gene** | *TaAOS* | *TaHPL* | *TaKSL* | *TaAAO* | *TaREC* | *TaBG* | *TaMAPK* |  |
| --- | --- | --- | --- | --- | --- | --- | --- | --- |
| *T. aestivum cv. Chinese spring* | 4B:495147007-495149756 | 6D:13059842-13062314 | Un:12441012-12441346 | 7B:687590126-687591793 | 2D:37645390-37646533 | 3B:77171542-771172472 | 4A:120605915-120606435 |  |
| *A. tauschii* | 4D:408912584-408914208 | 6D:12439081-12441898 | 2D:11789605-11789886 | 7D:610346598-610348258 | 2D:39227325-39228468 | 3D:588834922-588835850 | 4D:352873902-352874421 |  |
| *H. vulgare* | 4H:551553544-551555019 | 6D:12439081-12441898 | / | 7H:638692826-638694028 | 4H:463076331-463076591 | 3H:669231564-669232045 | 4H:480894229-480894749 |  |
| *O. sativa* | 3:6608878-6609307 | 2:555800-556066 | 4:5430598-5430721 | / | 3:10419228-10419521 | 1:41365909-41366298 | 3:9848881-9849220 |  |
| *Z. mays* | 1:29125887-29126242 | 4:245807235-245807497 | / | / | 1:46498895-46499188 | 3:153225680-153225857 | 9:141028451-141028896 |  |
|  |  |  |  |  |  |  |  |  |
| **Species/gene** | *TaCDPK* | *TaCYP450* | *TaNCED* | *TaABI* | *TaPIMP* | *TaRBOH* | *TaZEP* |  |
| *T. aestivum cv. Chinese spring* | 2D:161373811-161376953 | 7D:258236253-258238309 | 5A:572021505-572023279 | 3B:378687185-378688024 | 1B:615731797-615736596 | 3A:509419957-509424756 | 2A:542649865-542654664 |  |
| *A. tauschii* | 2D:162877394-162880536 | 7D:260079972-260082028 | 5D:461594567-461596180 | 3D:291555434-291556273 | 1D:456628310-456628952 | 3D:395932717-395933514 | 2D:403352290-403353197 |  |
| *H. vulgare* | 2H:186437313-186437726 | 7H:243843973-243844915 | 5H:589662659-589664370 | 3H:360097164-360097638 | 1H:522193739-522194042 | 3H:528609600-528610140 | 2H:570917983-570918742 |  |
| *O. sativa* | 7:22861699-22861962 | 8:274222124-27422381 | 3:24959628-24960996 | 1:22618746-22618897 | 5:26988130-26988258 | 1:30632735-30632942 | 4:22374018-22374264 |  |
| *Z. mays* | 2:212773115-212773455 | 1:210345242-210345520 | 1:255021702-255023080 | 3:224588858-224589031 | 6:166302733-166302865 | 3:203440771-203441051 | 2:46298949-46299167 |  |
