# Supplementary material for: The modulation of stomatal conductance and photosynthetic parameters is involved in Fusarium head blight resistance in wheat
Source: PLoS One. 2020 Jun 30;15(6):e0235482. doi: 10.1371/journal.pone.0235482 (PMC7326183; doi:10.1371/journal.pone.0235482)
Supplement: S3 Table — The E and R2 values were calculated as follows: five 1:10 serial dilutions (1:1–1:10000) were obtained for each sample of cDNA and amplified in four technical replicates obtained from four independent biological replicates. E and R2 values were calculated by the slope of the standard curve obtained by plotting fluorescence versus serial dilution concentrations using the equation E=10(−1slope)−1. The E and R2 values for the TaTUB taken from Rebelde were not calculated (x), because amplification did not occur at 1:100, 1:1000 and 1:10000 cDNA dilutions. (DOCX) [file pone.0235482.s003.docx]

**S3 Table**

|  | **Rebelde** | | **Sumai3** | |
| --- | --- | --- | --- | --- |
| **Gene** | **E** | **R^2^** | **E** | **R^2^** |
| *TaAOS* | 1,0633 | 0,9972 | 1,1191 | 0,9967 |
| *TaHPL* | 1,0336 | 0,9970 | 0,9759 | 0,9920 |
| *TaKSL* | 1,1207 | 0,9972 | 1,1094 | 0,9985 |
| *TaAAO* | 1,0747 | 0,9967 | 1,0643 | 0,9953 |
| *TaREC* | 0,9787 | 0,9982 | 1,2463 | 0,9911 |
| *TaBG* | 1,0967 | 0,9907 | 1,1484 | 0,9965 |
| *TaMAPK* | 0,9022 | 0,9955 | 0,9763 | 0,9954 |
| *TaCDPK* | 1,0815 | 0,9967 | 0,9606 | 0,9949 |
| *TaCYP450* | 1,0485 | 0,9925 | 1,0854 | 0,9925 |
| *TaNCED* | 1,1080 | 0,9944 | 1,0873 | 0,9922 |
| *TaABI* | 1,1746 | 0,9972 | 0,9012 | 0,9957 |
| *TaPIMP* | 1,1826 | 0,9934 | 1,0508 | 0,9939 |
| *TaRBOH* | 0,9844 | 0,9954 | 0,9462 | 0,9969 |
| *TaZEP* | 1,1052 | 0,9984 | 1,0785 | 0,9891 |
| *TaGAPDH* | 1,0249 | 0,9842 | 1,0354 | 0,9857 |
| *TaPR1* | 0,9056 | 0,9956 | 1,0893 | 0,9965 |
| *TaACT* | 1,1748 | 0,9954 | 1,1694 | 0,9961 |
| *TaTUB* | x | x | 1,0448 | 0,9849 |
| *TaFNR* | 0,8988 | 0,9835 | 0,9249 | 0,9661 |
