# Supplementary material for: The modulation of stomatal conductance and photosynthetic parameters is involved in Fusarium head blight resistance in wheat
Source: PLoS One. 2020 Jun 30;15(6):e0235482. doi: 10.1371/journal.pone.0235482 (PMC7326183; doi:10.1371/journal.pone.0235482)
Supplement: S4 Table — Cq values were calculated from four technical replicates derived from four independent biological replicates. TaTUB from Rebelde did not occur at 1:100, 1:1000 and 1:10000 cDNA dilutions, so no Cq values were detected (nd), therefore, the SE was not calculated (x). (DOCX) [file pone.0235482.s004.docx]

**S4 Table**

|  |  | **Rebelde** | | **Sumai3** | |
| --- | --- | --- | --- | --- | --- |
| **Reference gene** | **Dilution** | **Cq** | **SE (±)** | **Cq** | **SE (±)** |
| *TaACT* | 1:1 | 19,51 | 0,194 | 19,63 | 0,348 |
|  | 1:10 | 22,20 | 0,211 | 22,38 | 0,342 |
|  | 1:100 | 24,98 | 0,242 | 25,15 | 0,343 |
|  | 1:1000 | 27,79 | 0,266 | 28,00 | 0,311 |
|  | 1:10000 | 31,53 | 0,246 | 31,69 | 0,357 |
| *TaTUB* | 1:1 | 36,65 | 0,638 | 19,54 | 1,366 |
|  | 1:10 | 39,51 | 0,770 | 23,08 | 1,794 |
|  | 1:100 | nd | x | 25,22 | 1,440 |
|  | 1:1000 | nd | x | 30,11 | 1,570 |
|  | 1:10000 | nd | x | 32,12 | 1,966 |
| *TaFNR* | 1:1 | 12,07 | 2,563 | 13,45 | 2,122 |
|  | 1:10 | 14,05 | 2,014 | 16,69 | 2,665 |
|  | 1:100 | 17,73 | 2,962 | 18,26 | 2,135 |
|  | 1:1000 | 22,62 | 2,136 | 22,91 | 2,352 |
|  | 1:10000 | 25,74 | 2,336 | 27,92 | 2,235 |
