# Supplementary material for: The modulation of stomatal conductance and photosynthetic parameters is involved in Fusarium head blight resistance in wheat
Source: PLoS One. 2020 Jun 30;15(6):e0235482. doi: 10.1371/journal.pone.0235482 (PMC7326183; doi:10.1371/journal.pone.0235482)
Supplement: S5 Table — Relative expression values were obtained by using the equation 2-ΔΔCq with TaACT as a reference gene and mock treatments used to normalize the relative expression levels. The data represent averages and standard errors for the four independent biological replicates and the four technical replicates examined. The data were subjected to two-way analysis of variance (ANOVA), where the two independent variables were the wheat genotype (Rebelde and Sumai3) and the plant treatment (drought stress, 24 hpi, 48 hpi, 72 hpi and 10 dpi) while the dependent variable was the relative expression level. Different letters among the plant treatments and wheat genotypes were assessed by means of significant F values by applying the Tukey Honestly Significant Difference test (Tukey test) at 0.99 confidence level (P<0.01). (DOCX) [file pone.0235482.s005.docx]

**S5 Table**

|  |  |  |  |  |  |  |  |  |  |  |  |
| --- | --- | --- | --- | --- | --- | --- | --- | --- | --- | --- | --- |
|  |  | **Drought stress** | | **24 hpi** | | **48 hpi** | | **72 hpi** | | **10 dpi** | |
| **Gene** |  | **Rebelde** | **Sumai3** | **Rebelde** | **Sumai3** | **Rebelde** | **Sumai3** | **Rebelde** | **Sumai3** | **Rebelde** | **Sumai3** |
| *TaAOS* | Relative expression | 0,298 | 0,481 | 0,293 | 2,672 | 0,392 | 3,178 | 0,181 | 1,567 | 0,161 | 1,603 |
|  | Standard error (±) | 0,028 | 0,093 | 0,032 | 0,783 | 0,061 | 0,219 | 0,029 | 0,285 | 0,034 | 0,306 |
|  | *P<0,01* | cd | cd | cd | ab | d | a | d | bc | d | bc |
| *TaHPL* | Relative expression | 0,510 | 1,091 | 0,515 | 7,895 | 0,319 | 12,556 | 0,268 | 3,246 | 0,009 | 0,717 |
|  | Standard error (±) | 0,016 | 0,038 | 0,012 | 1,550 | 0,026 | 0,602 | 0,025 | 0,131 | 0,003 | 0,204 |
|  | *P<0,01* | d | cd | d | b | d | a | d | c | d | cd |
| *TaKSL* | Relative expression | 0,372 | 0,279 | 0,592 | 2,621 | 1,617 | 2,021 | 0,719 | 0,710 | 0,401 | 1,025 |
|  | Standard error (±) | 0,084 | 0,091 | 0,190 | 0,593 | 0,344 | 0,357 | 0,023 | 0,230 | 0,069 | 0,592 |
|  | *P<*0,01 | b | b | b | ab | ab | ab | b | b | b | b |
| *TaAAO* | Relative expression | 0,036 | 0,266 | 0,038 | 1,702 | 0,036 | 1,290 | 0,026 | 0,178 | 0,013 | 0,202 |
|  | Standard error (±) | 0,005 | 0,017 | 0,003 | 0,460 | 0,005 | 0,085 | 0,009 | 0,023 | 0,007 | 0,045 |
|  | *P<*0,01 | c | b | c | a | c | a | c | bc | c | bc |
| *TaREC* | Relative expression | 0,381 | 0,221 | 1,048 | 1,300 | 0,361 | 4,197 | 0,322 | 0,259 | 0,213 | 0,172 |
|  | Standard error (±) | 0,075 | 0,009 | 0,091 | 0,346 | 0,017 | 1,263 | 0,014 | 0,015 | 0,044 | 0,054 |
|  | *P<*0,01 | b | b | b | b | b | a | b | b | b | b |
| *TaBG* | Relative expression | 0,987 | 4,313 | 1,302 | 70,644 | 0,941 | 50,534 | 0,919 | 25,137 | 5,668 | 8,452 |
|  | Standard error (±) | 0,056 | 0,418 | 0,154 | 19,553 | 0,013 | 10,159 | 0,317 | 2,994 | 1,974 | 4,575 |
|  | *P<*0,01 | b | b | b | a | b | a | b | b | b | b |
| *TaMAPK* | Relative expression | 0,713 | 0,754 | 0,555 | 7,376 | 1,412 | 6,129 | 1,098 | 1,993 | 3,742 | 1,572 |
|  | Standard error (±) | 0,088 | 0,107 | 0,072 | 1,113 | 0,113 | 0,313 | 0,042 | 0,021 | 0,821 | 0,214 |
|  | *P<*0,01 | c | c | c | a | c | a | c | bc | b | bc |
| *TaCDPK* | Relative expression | 0,623 | 0,808 | 0,614 | 2,321 | 0,650 | 2,494 | 0,545 | 0,465 | 0,458 | 0,588 |
|  | Standard error (±) | 0,052 | 0,026 | 0,079 | 0,471 | 0,073 | 0,129 | 0,030 | 0,240 | 0,091 | 0,094 |
|  | *P<*0,01 | b | b | b | a | b | a | b | b | b | b |
| *TaCYP450* | Relative expression | 0,920 | 1,743 | 0,885 | 39,768 | 0,657 | 53,172 | 0,372 | 7,589 | 0,029 | 5,126 |
|  | Standard error (±) | 0,069 | 0,517 | 0,095 | 11,314 | 0,073 | 6,406 | 0,066 | 0,958 | 0,014 | 1,594 |
|  | *P<*0,01 | b | b | b | a | b | a | b | b | b | b |
| *TaNCED* | Relative expression | 0,663 | 0,597 | 0,636 | 2,732 | 0,721 | 3,315 | 0,702 | 0,730 | 0,105 | 0,663 |
|  | Standard error (±) | 0,071 | 0,106 | 0,032 | 0,441 | 0,044 | 0,615 | 0,034 | 0,026 | 0,012 | 0,115 |
|  | *P<*0,01 | b | b | b | a | b | a | b | b | b | b |
| *TaABI* | Relative expression | 0,661 | 0,378 | 0,522 | 5,791 | 1,044 | 5,979 | 0,604 | 1,175 | 0,070 | 1,094 |
|  | Standard error (±) | 0,044 | 0,222 | 0,094 | 1,399 | 0,034 | 0,701 | 0,031 | 0,176 | 0,011 | 0,084 |
|  | *P<*0,01 | b | b | b | a | b | a | b | b | b | b |
| *TaPIMP* | Relative expression | 0,916 | 0,004 | 0,818 | 1,918 | 1,221 | 2,409 | 0,462 | 0,352 | 0,019 | 0,368 |
|  | Standard error (±) | 0,046 | 0,000 | 0,092 | 0,469 | 0,117 | 0,008 | 0,048 | 0,020 | 0,003 | 0,124 |
|  | *P<*0,01 | bc | d | bc | a | b | a | cd | cd | d | cd |
| *TaRBOH* | Relative expression | 0,371 | 1,073 | 0,547 | 1,007 | 0,472 | 1,152 | 0,199 | 0,226 | 0,038 | 0,441 |
|  | Standard error (±) | 0,021 | 0,031 | 0,026 | 0,021 | 0,040 | 0,097 | 0,009 | 0,026 | 0,026 | 0,074 |
|  | *P<*0,01 | bc | a | b | a | b | a | cd | cd | d | b |
| *TaZEP* | Relative expression | 2,638 | 1,533 | 0,838 | 7,708 | 1,314 | 14,446 | 1,032 | 1,657 | 0,035 | 1,310 |
|  | Standard error (±) | 0,232 | 0,107 | 0,049 | 2,757 | 0,126 | 2,672 | 0,085 | 0,236 | 0,003 | 0,073 |
|  | *P<*0,01 | c | c | c | b | c | a | c | c | c | c |
| *TaGADPH* | Relative expression  Standard error (±)  *P<*0,01 | 1,238  0,410  b | 1,462  0,157  b | 0,899  0,014  b | 1,579  0,324  b | 1,025  0,346  b | 2,284  0,237  a | 1,147  0,411  b | 2,467  0,222  a | 1,053  0,154  b | 3,027  0,263  a |
| *TaPR1* | Standard error (±) | 0,816 | 55,270 | 1,444 | 23,705 | 2,133 | 65,303 | 4,337 | 1,426 | 9,392 | 38,874 |
|  | *P<*0,01 | 0,055 | 1,910 | 0,286 | 1,201 | 0,132 | 7,020 | 0,756 | 0,154 | 1,155 | 2,036 |
|  | *P<*0,01 | e | b | e | d | e | a | e | e | e | c |
