# Supplementary figures and images for: The modulation of stomatal conductance and photosynthetic parameters is involved in Fusarium head blight resistance in wheat
Source: PLoS One. 2020 Jun 30;15(6):e0235482. doi: 10.1371/journal.pone.0235482 (PMC7326183; doi:10.1371/journal.pone.0235482)

**S1 Figure**


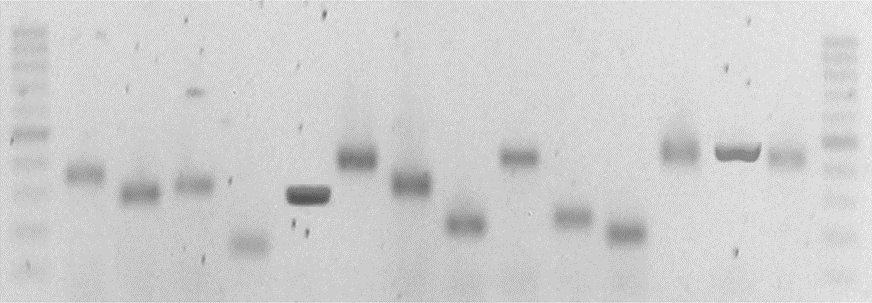


M 1 2 3 4 5 6 7 8 9 10 11 12 13 14 M

100 bp-

200 bp-

300 bp-

400 bp-

500 bp-


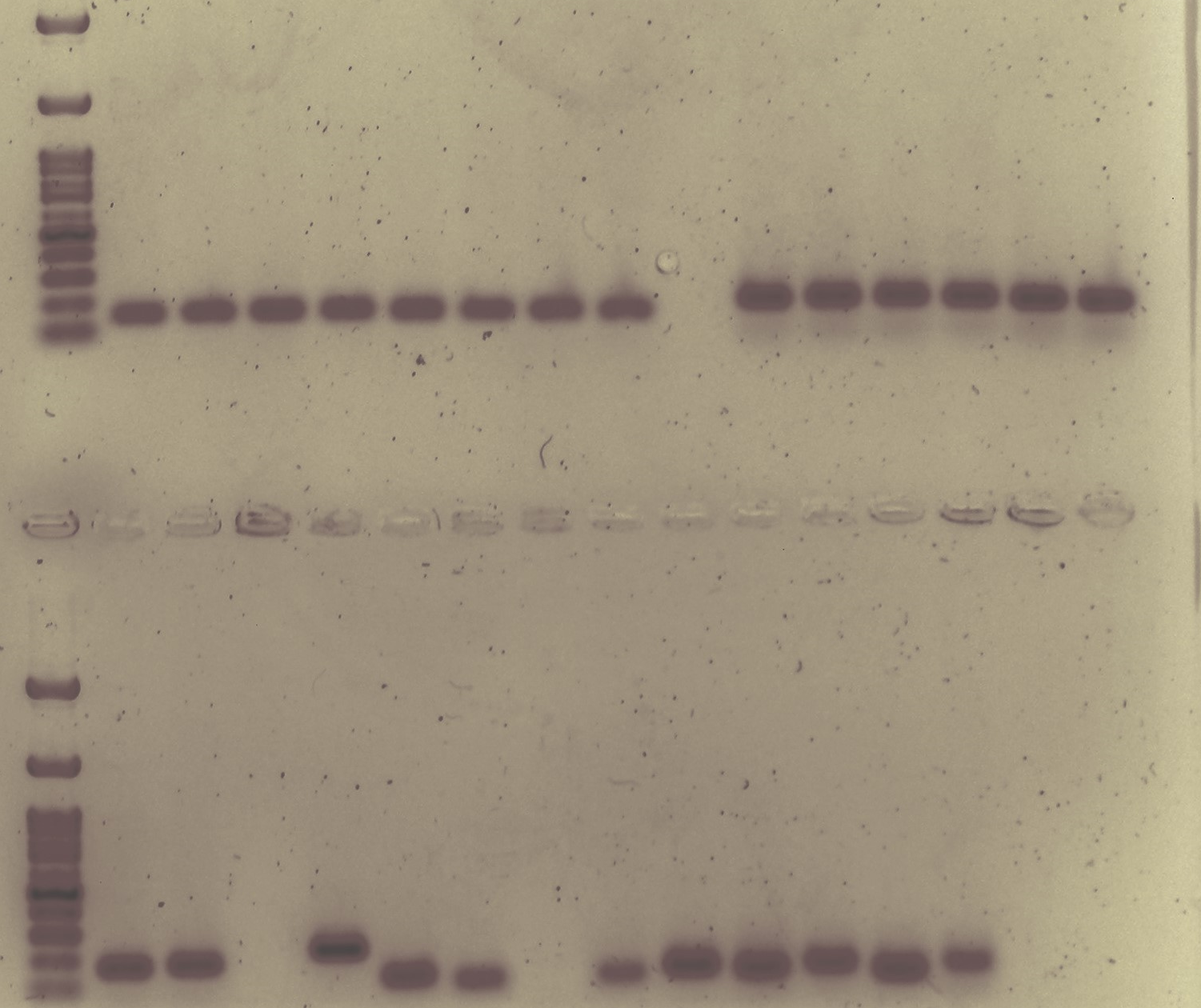

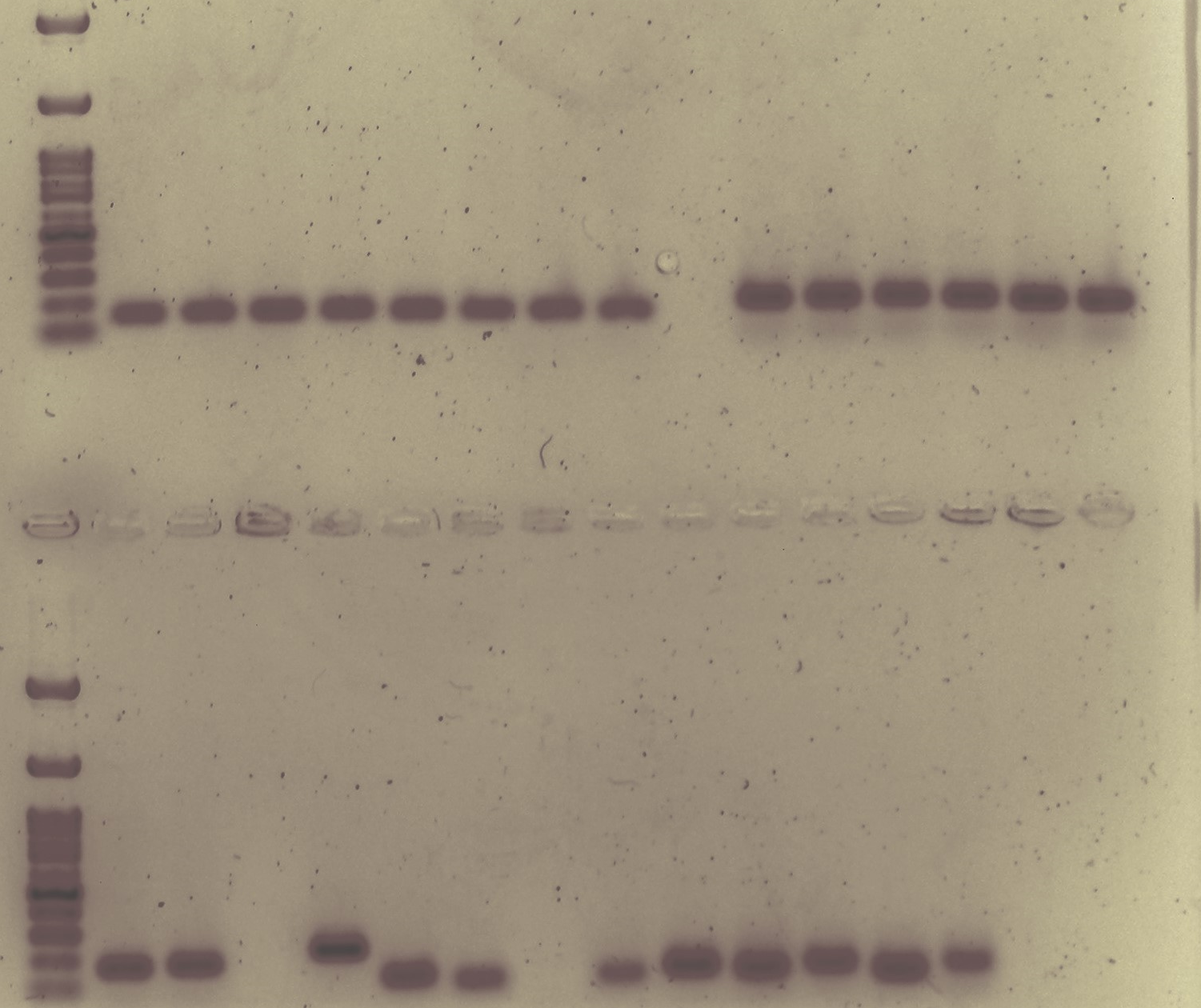


M 15 M 16


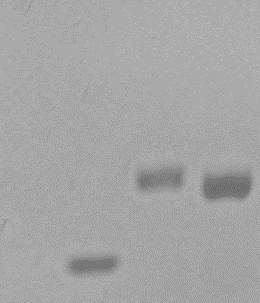

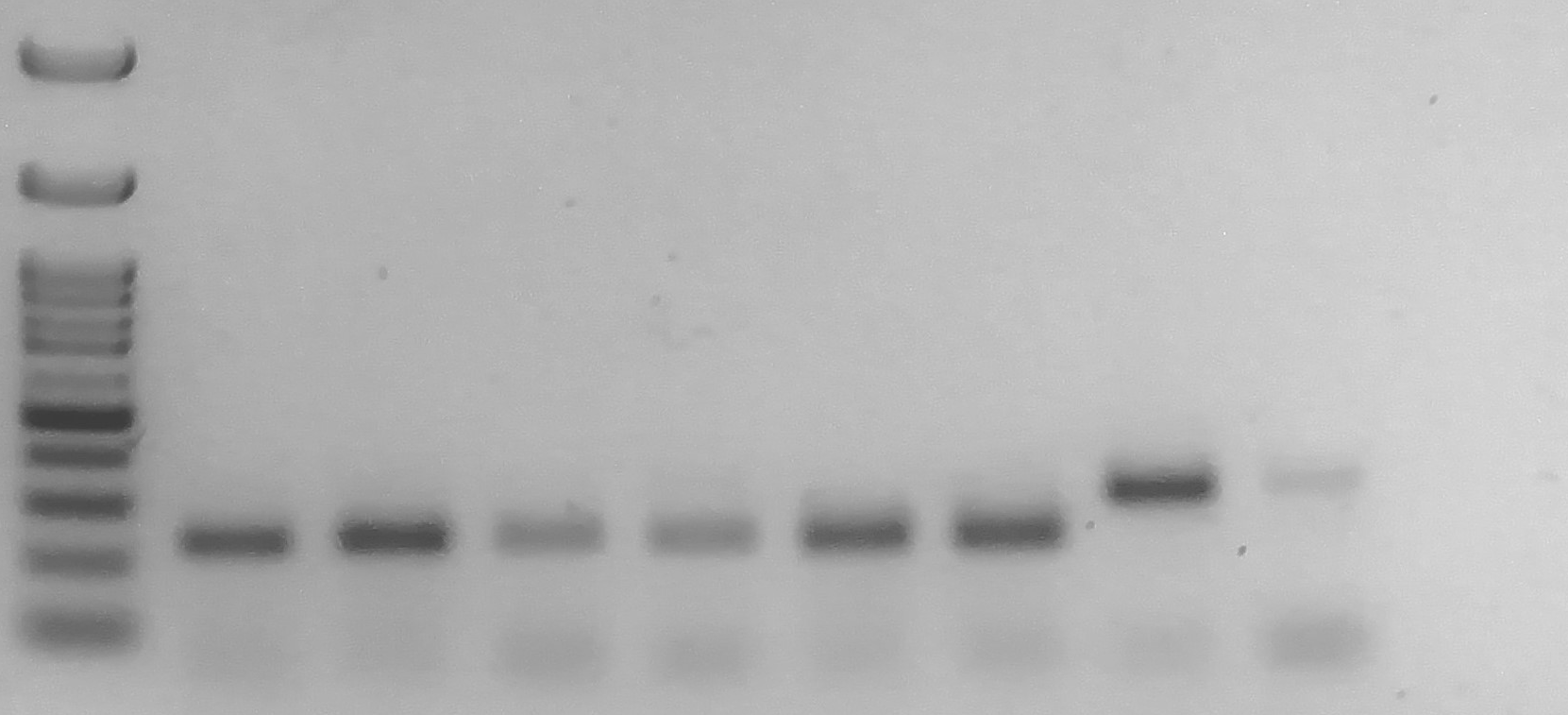


M 17 18 19 20

M 21

**
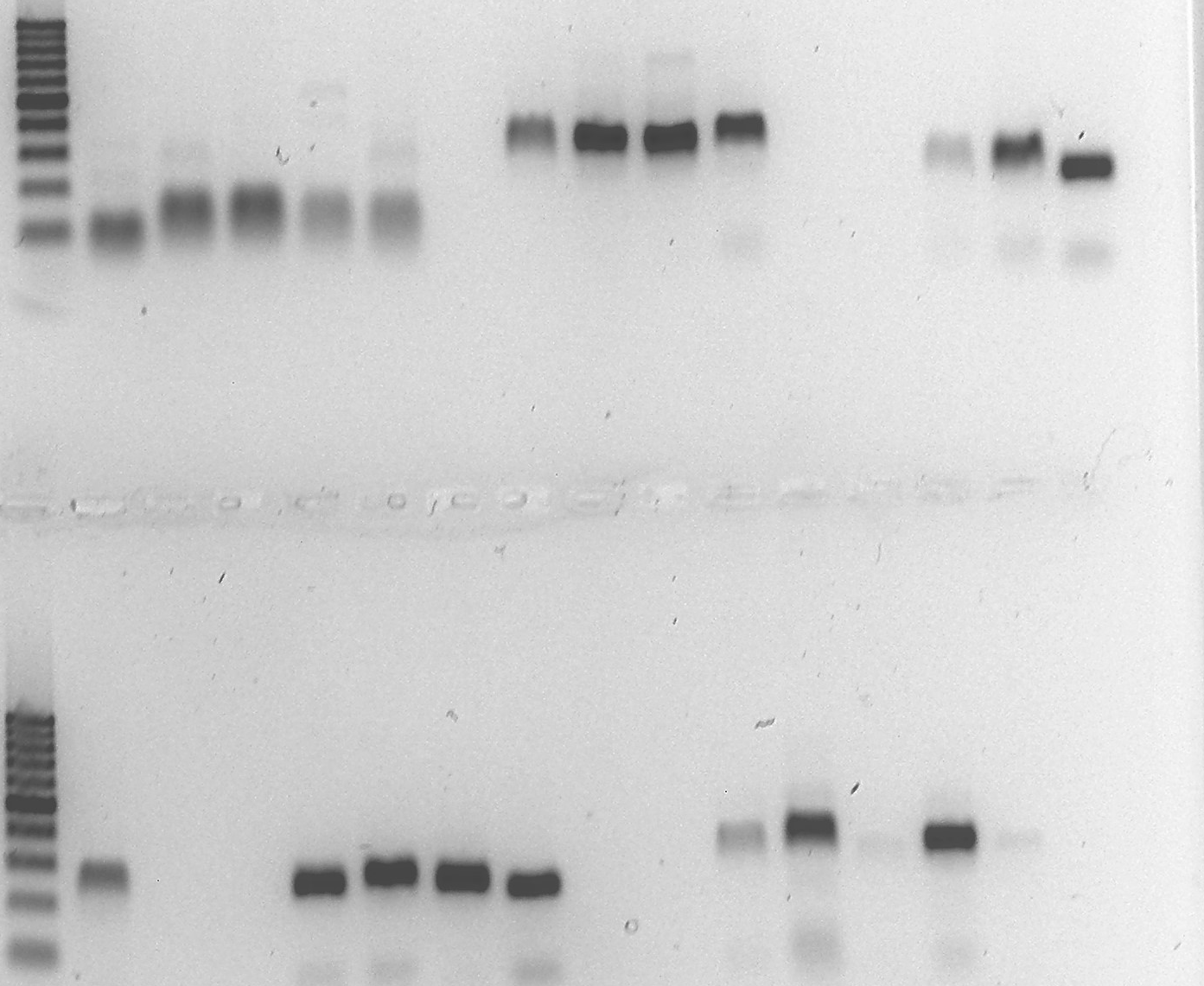
**

Supplement: S1 Fig — M) ExcelBand™ 100 bp + 3K DNA Ladder (Smobio), 1) TaAOS, 2) TaABI, 3) TaACT, 4) TaPR1, 5) TaKSL, 6) TaBG, 7) TaPIMP, 8) TaREC, 9) TaCYP450, 10) TaZEP, 11) TaHPL, 12) TaCDPK, 13) TaNCED, 14) TaRBOH, 15) TaAAO, 16) TaMAPK, 17) TaTUB from Rebelde (not detected), 18) TaTUB from Sumai3, 19) TaFNR from Rebelde, 20) TaFNR from Sumai3, 21) TaGAPDH from Rebelde. The Fig represents the original picture of the gel. (DOCX) [file pone.0235482.s006.docx]

**S2 Figure**

**
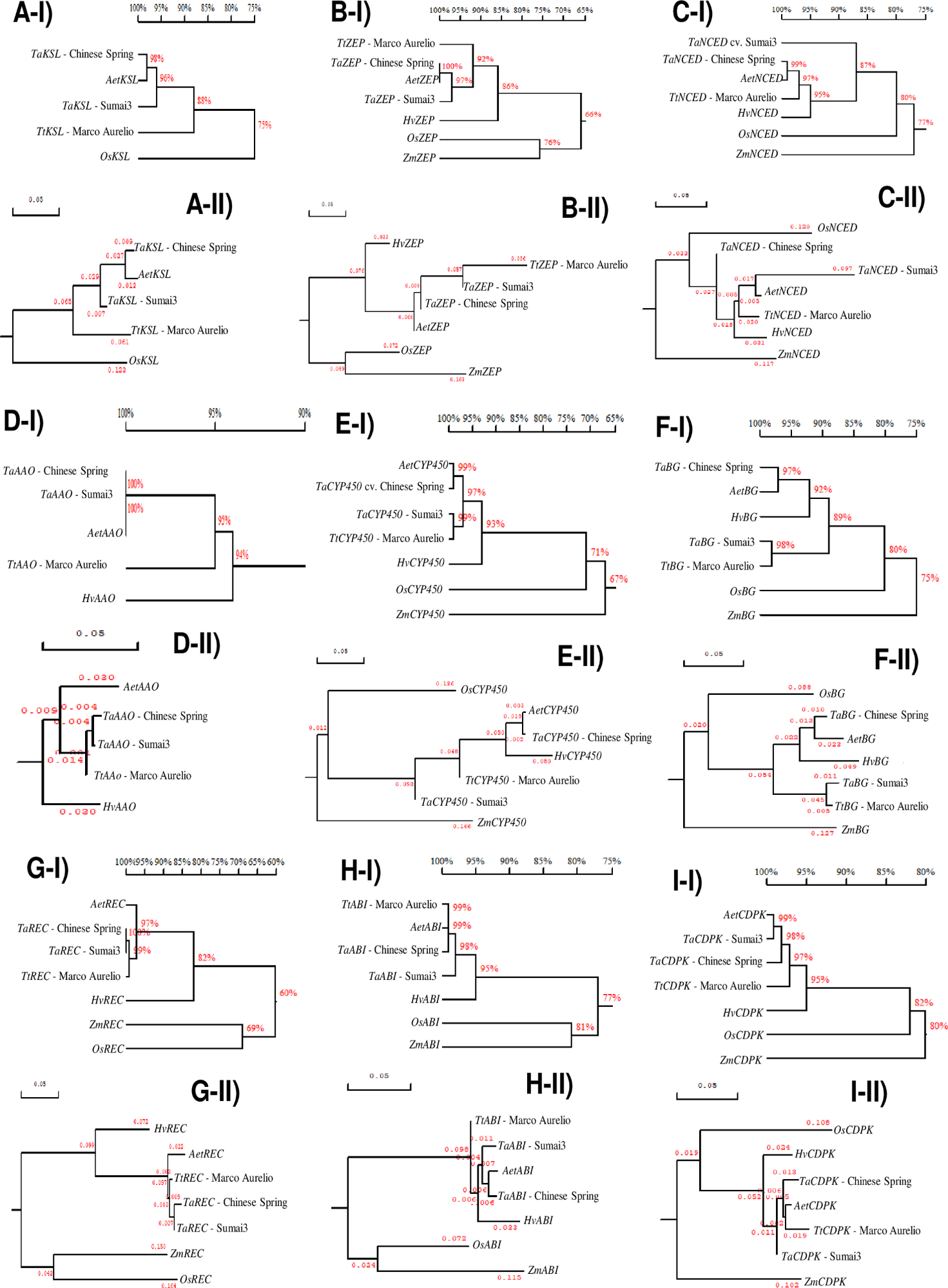
**

**
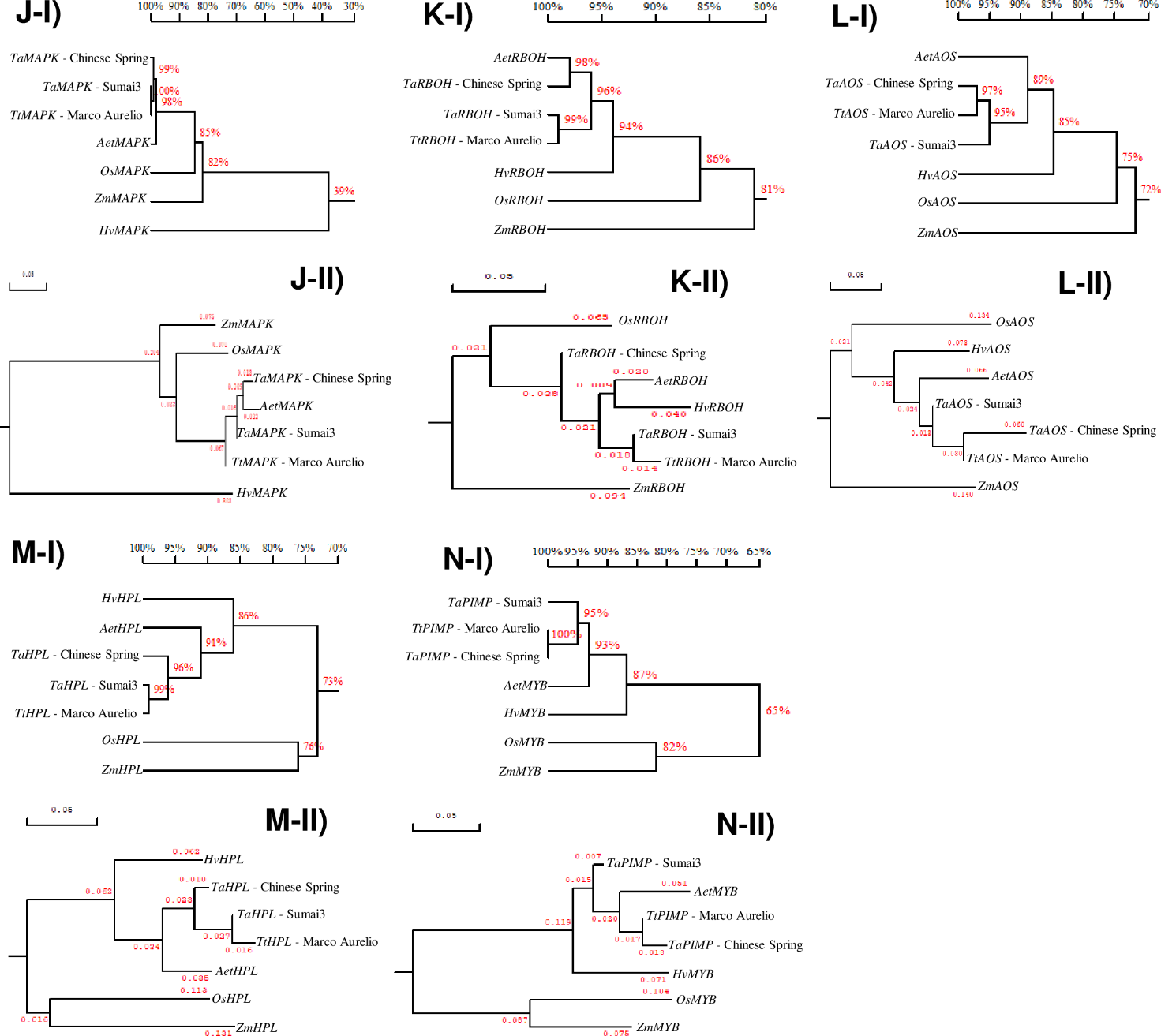
**

Supplement: S2 Fig — Homology trees are represented in Figs from A-I to N-I and phylogeny trees are represented in Figs from A-II to N-II. The trees were obtained by clustering nucleotide sequences isolated using the primer pairs listed in S1 Table. The trees were constructed using DNAMAN software (Lynnon Biosoft, Quebec, Canada). (DOCX) [file pone.0235482.s007.docx]

**S3 Figure**

TaKSL


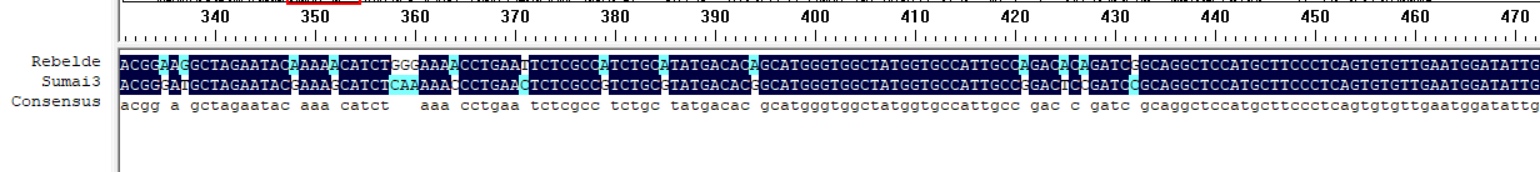


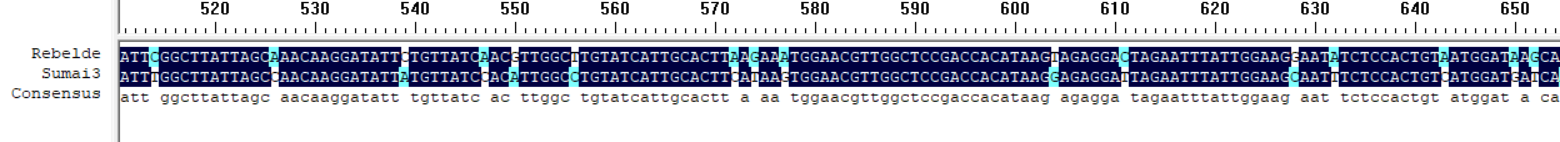


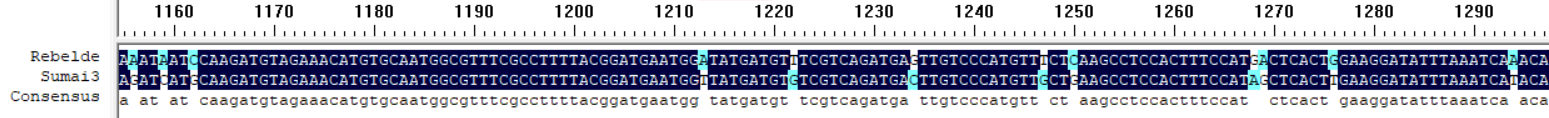


**
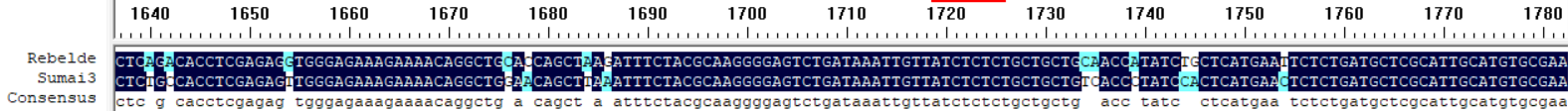
**

**
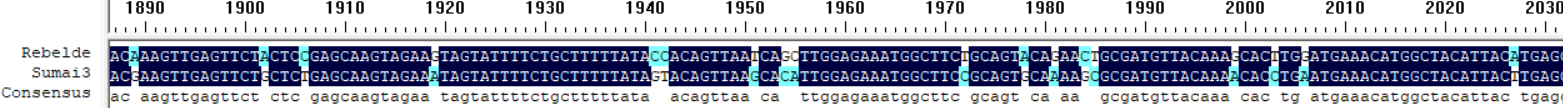
**

TaZEP

**
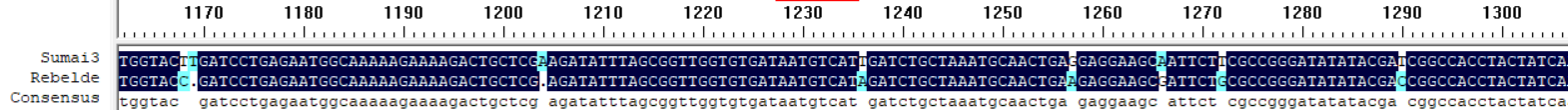
**

**
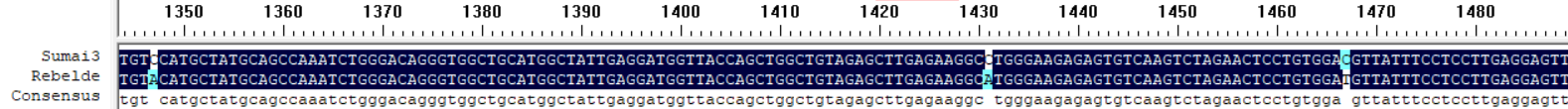
**

**
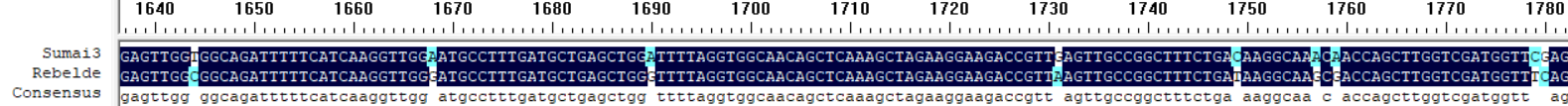
**

**
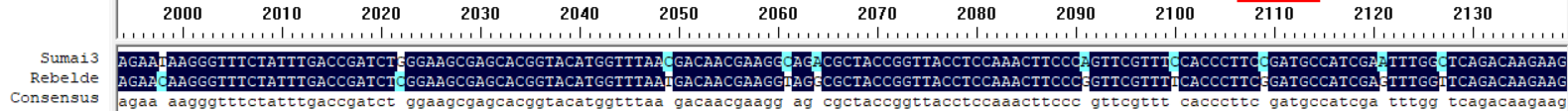
**

TaNCED

**
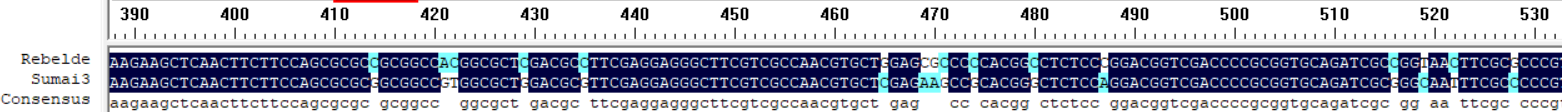
**

**
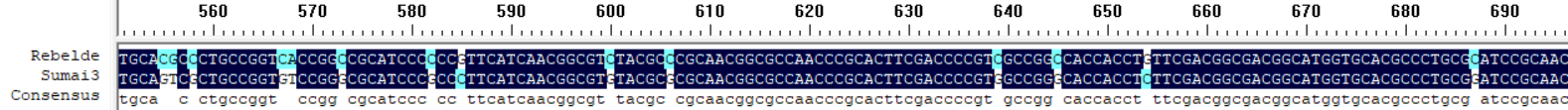
**

**
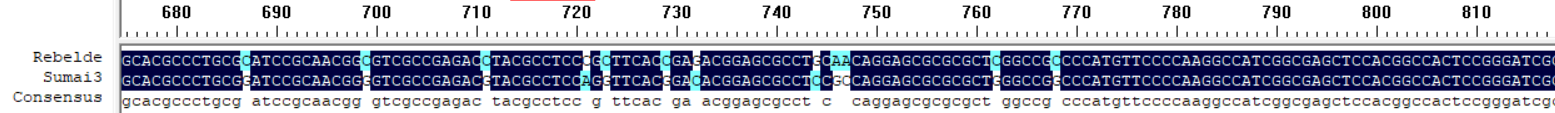
**

**
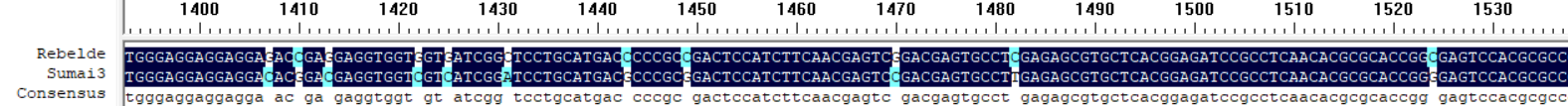
**

**
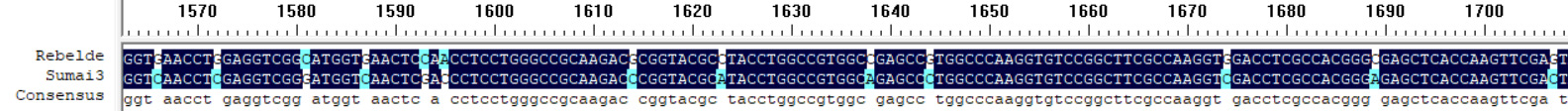
**

TaAAO

**
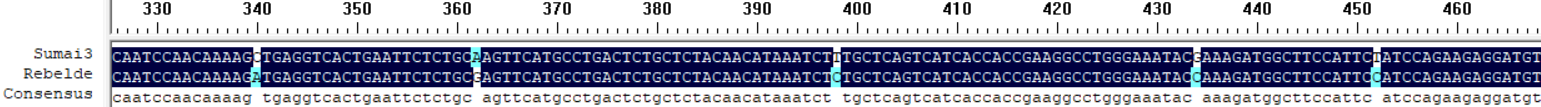
**

**
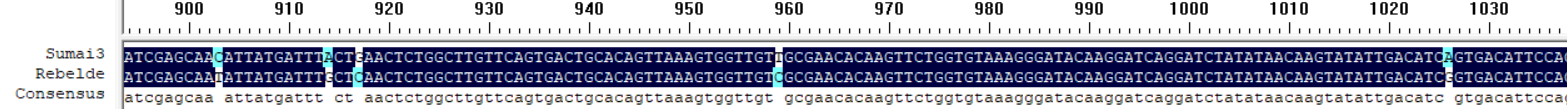
**

**
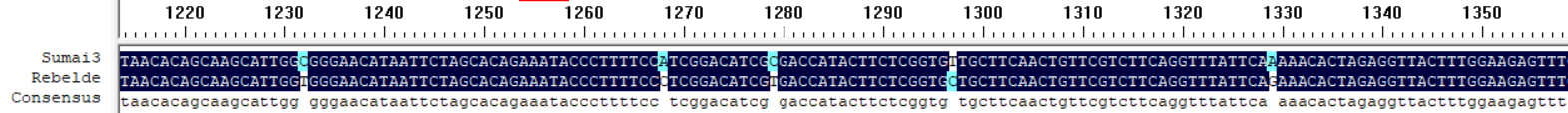
**

TaAOS


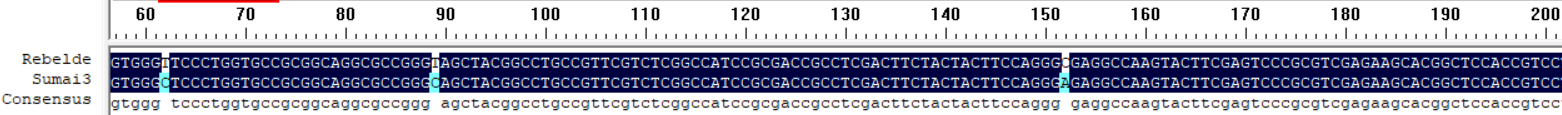


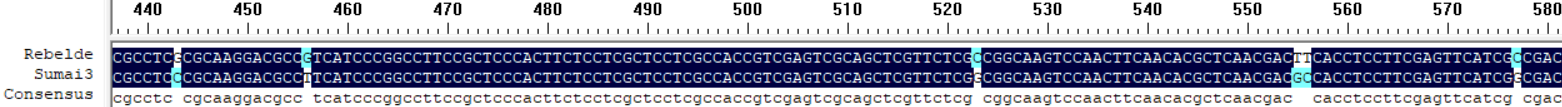


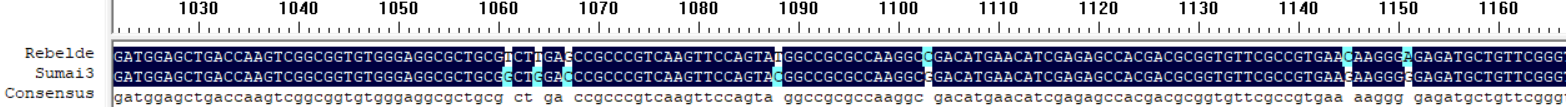


TaPIMP


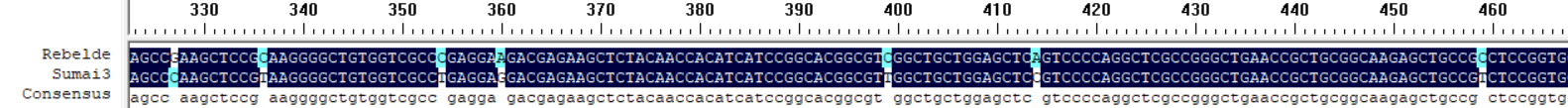


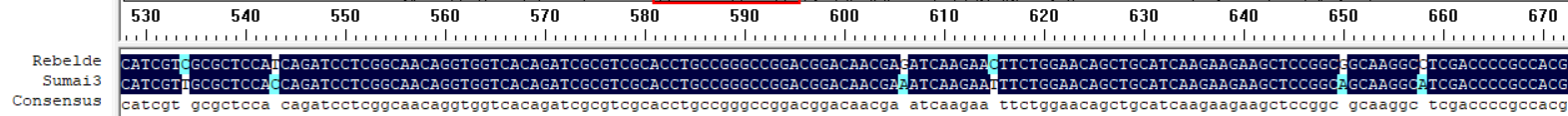


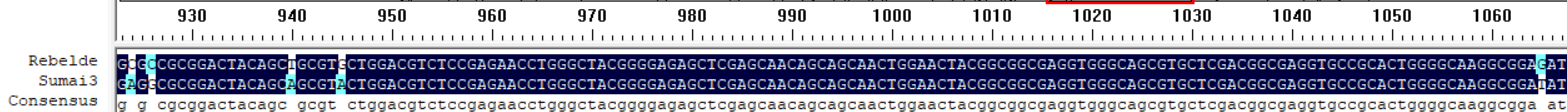

Supplement: S3 Fig — The sequences with a homology equal or less than 95% (TaKSL, TaZEP, TaNCED, TaAAO, TaAOS and TaPIMP) were aligned in order to observe the major pieces of the sequences characterized by SNPs. (DOCX) [file pone.0235482.s008.docx]

**S4 Figure**

Rebelde Sumai3


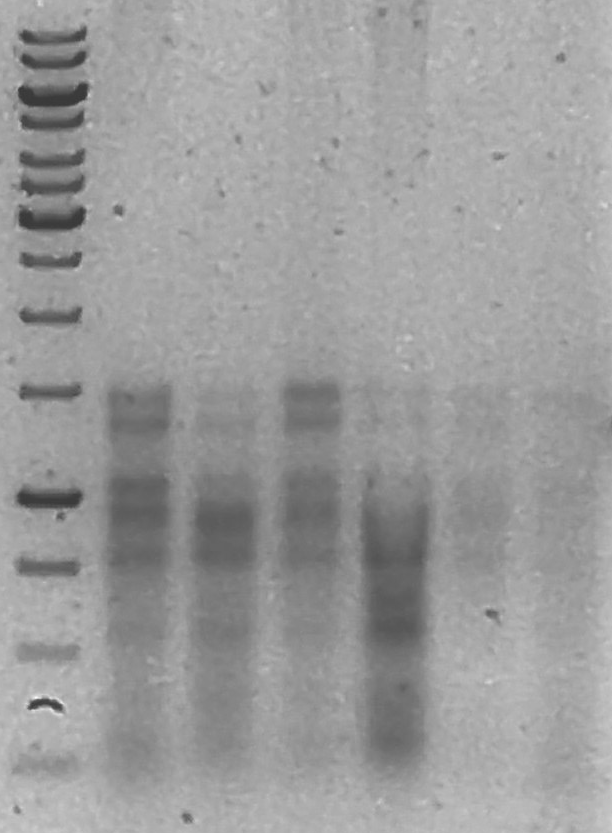

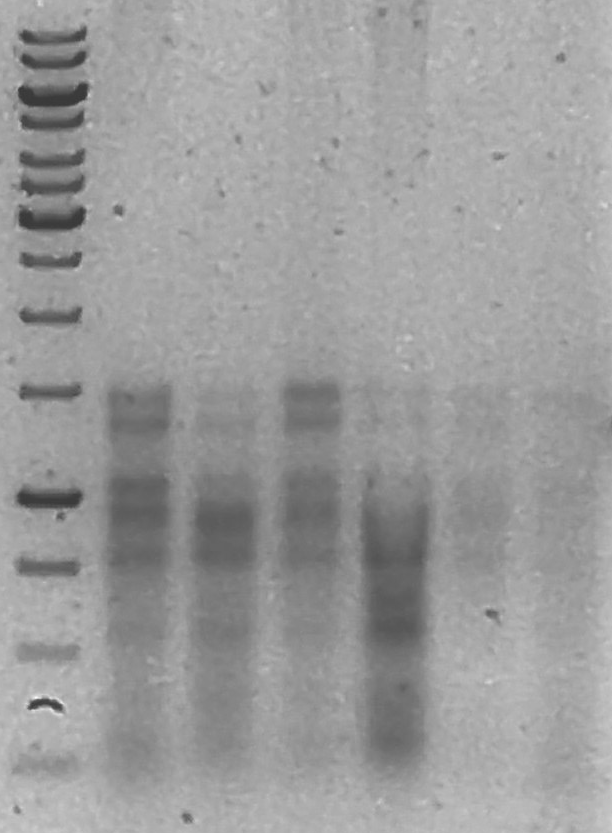


M 1 2 3 4 5 6 7 8


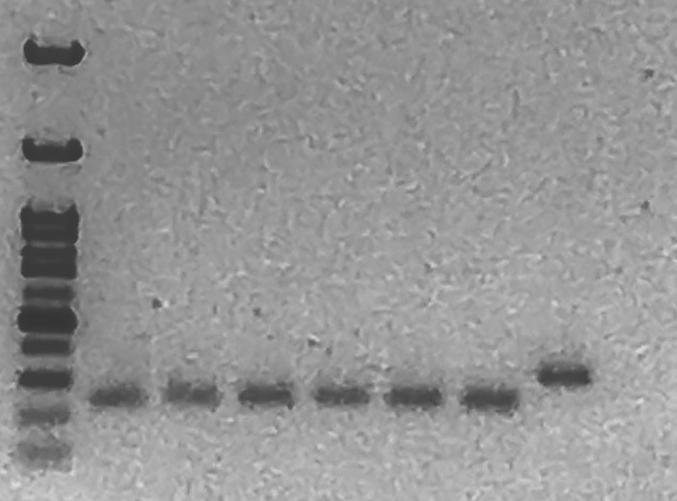


**A)**

**B)**

300 bp-

200 bp-


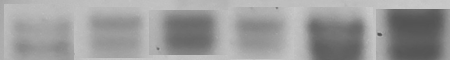

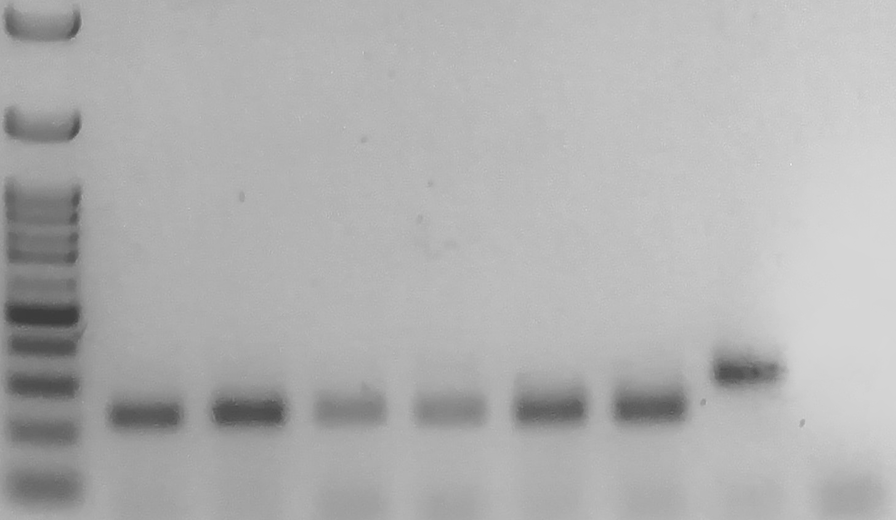


M 1 2 3 4 5 6 7 8

**C)**

**D)**

300 bp-

200 bp-

Supplement: S4 Fig — 1.5% agarose gel of total extracted RNA (A, C) and RT-PCR (B, D) from Rebelde and Sumai3, respectively. M) ExcelBand™ 100 bp + 3K DNA Ladder (Smobio), 1) Mock, 2) Drought stress, 3) 24 hpi, 4) 48 hpi, 5) 72 hpi, 6) 10 dpi, 7) Genomic DNA (gDNA) control, 8) No template control (NTC). The Fig represents the original picture of the gel. (DOCX) [file pone.0235482.s009.docx]

**S5 Figure**

**
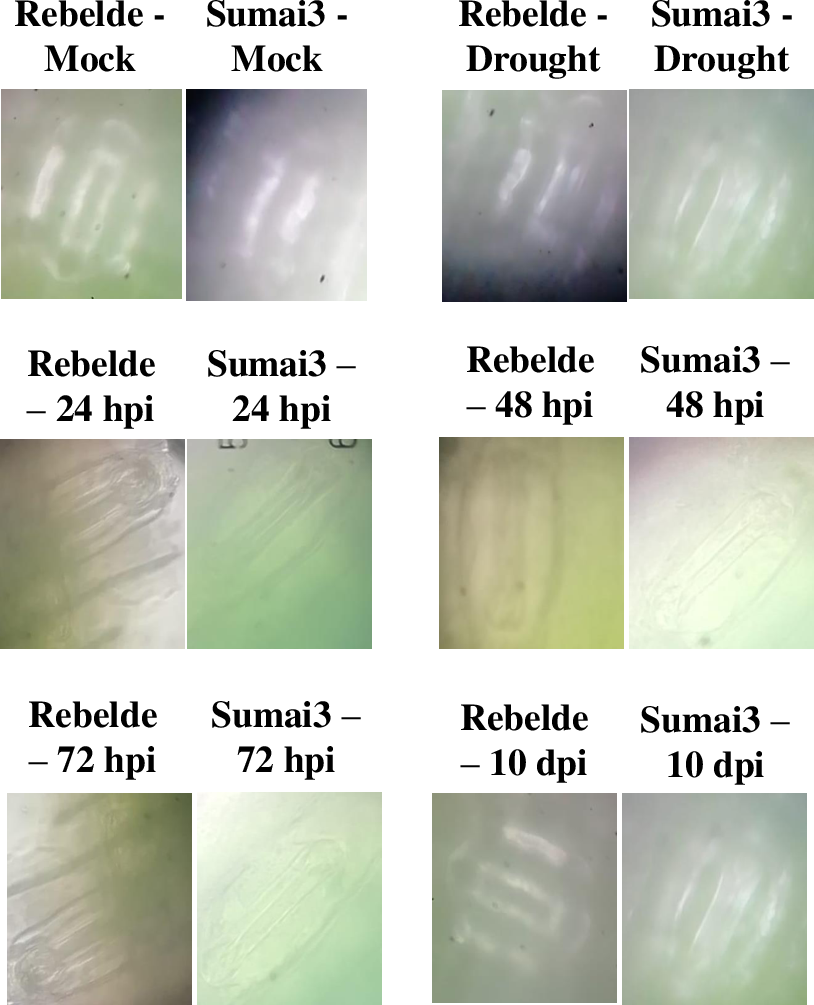
**

Supplement: S5 Fig — The pictures were obtained by observing a slice of the external glume with an optical microscope (Leitz Diaplan) and by using a magnification of 40X. (DOCX) [file pone.0235482.s010.docx]
