# Supplementary material for: The modulation of stomatal conductance and photosynthetic parameters is involved in Fusarium head blight resistance in wheat
Source: PLoS One. 2020 Jun 30;15(6):e0235482. doi: 10.1371/journal.pone.0235482 (PMC7326183; doi:10.1371/journal.pone.0235482)
Supplement: S1 Raw materials — (PDF) [file pone.0235482.s011.pdf]

Methodology used to acquire the pictures

The pictures were exposed to UV lights by using a transilluminator and the pictures were captured using a Nikon Coolpix 4500 Digital Camera. The raw images presented in this file were subjected to the following modifications only: the pictures were turned for straightening and only the external edges of the gel were cropped (all the wells of the gels (with and without a sample) are included in the pictures.

Original picture for the S1 Figure

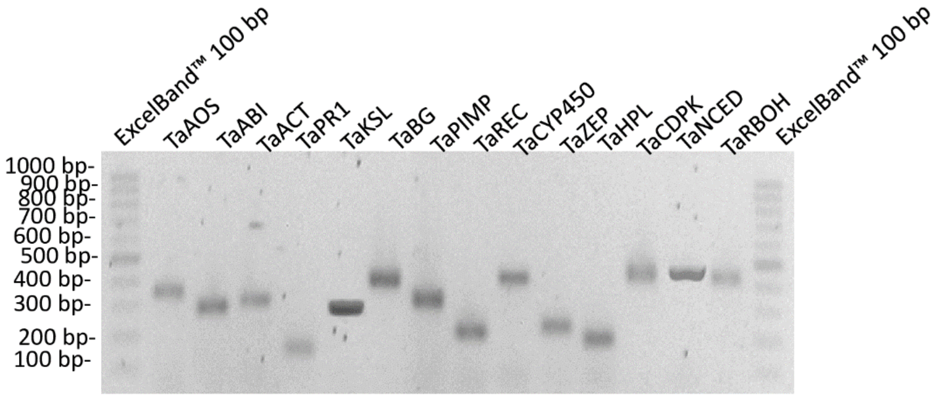

This picture was used to assemble S1 Figure (lanes 1-14) and is the original picture of the gel as presented in Supplementary materials. Only the edges of the gel were cropped.

Original picture for the S1 Figure

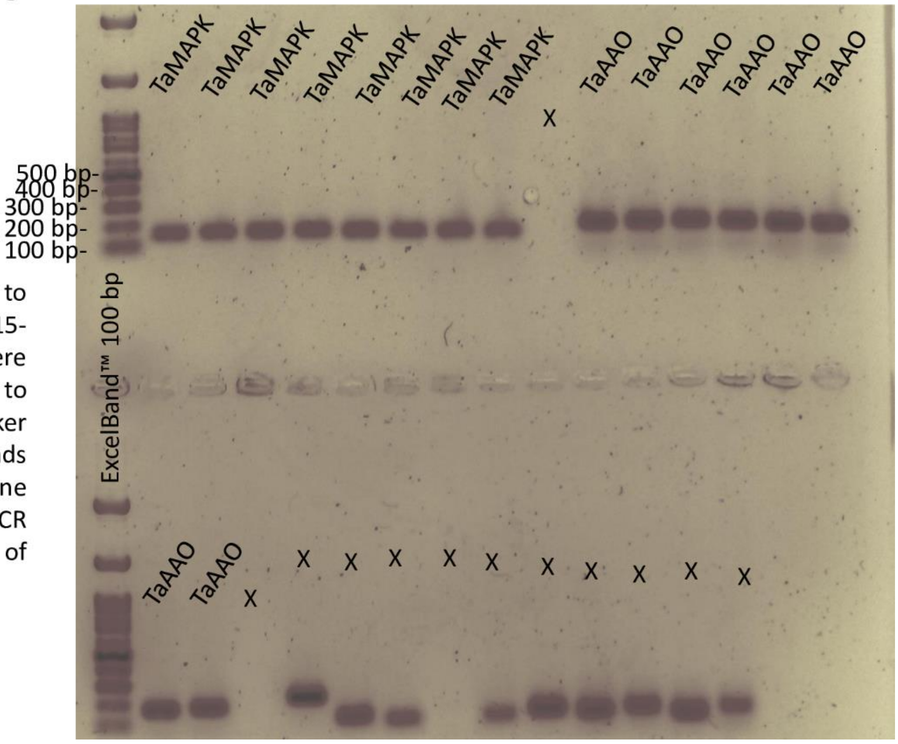

This picture was used to assemble S1 Figure (lanes 15-16). In the S1 Figure were inserted the lanes close to the marker and the marker itself. The other bands represent the same gene subjected to a gradient PCR (55-65°C). Only the edges of the gel were cropped.

Original picture for the S1 Figure

This picture was used to assemble S1 Figure (lanes 17-20). In the S1 Figure wi decided not to include the Ladder from this gel because it was not very clear. We used the ladder from the first picture included in this file. Only the edges of the gel were cropped.

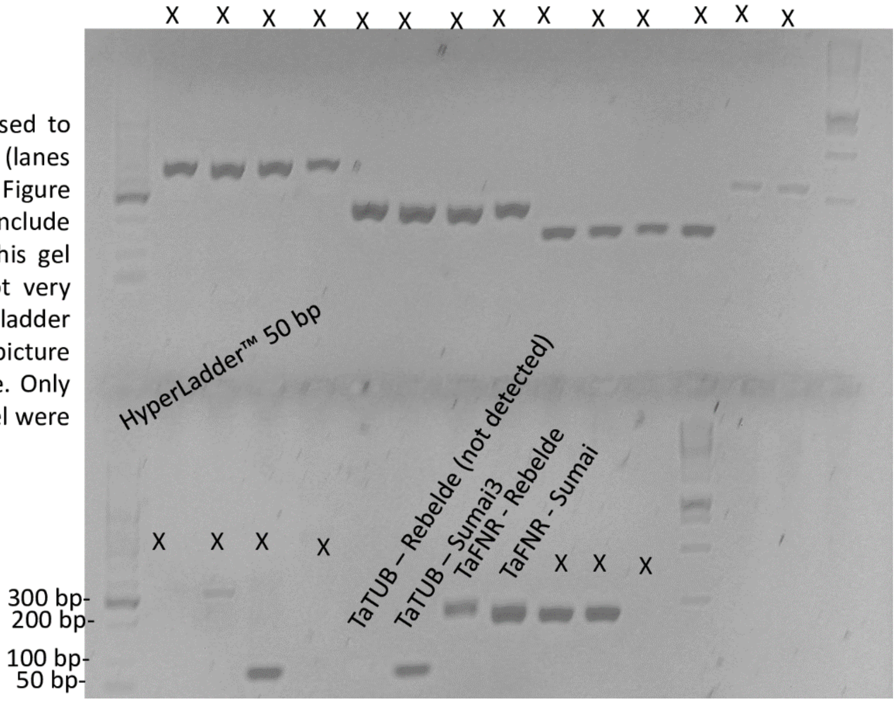

Original picture for the S1 Figure

300 bp-
200 bp-
100 bp-

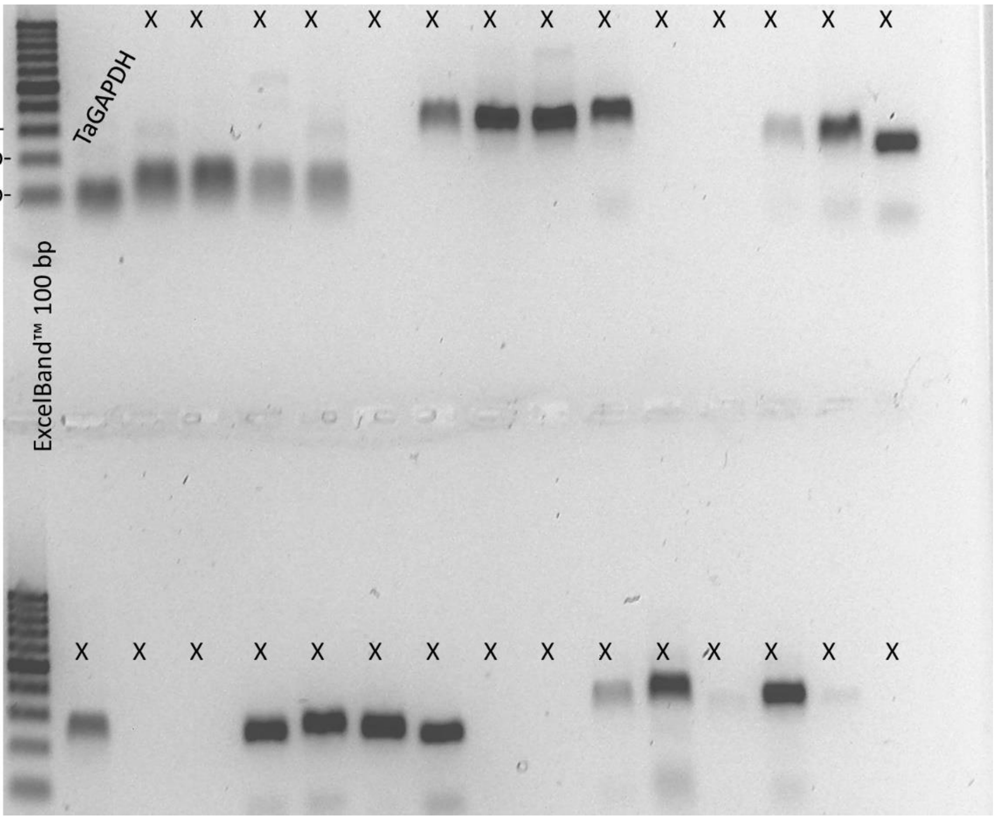

This picture was used to assemble S1 Figure (lane 21). Only the edges of the gel were cropped

Original pictures for the S4 Figure

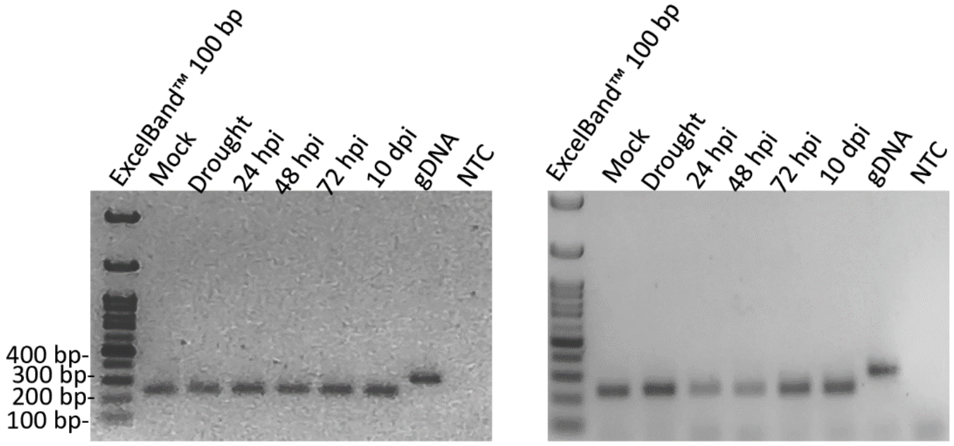

Rebelde

Sumai3

These pictures were used to assemble S4 Figure (B and D) and correspond to the original pictures as presented in Supplementary materials. The two pictures belong to two different gels and only the edges of the gels were cropped.

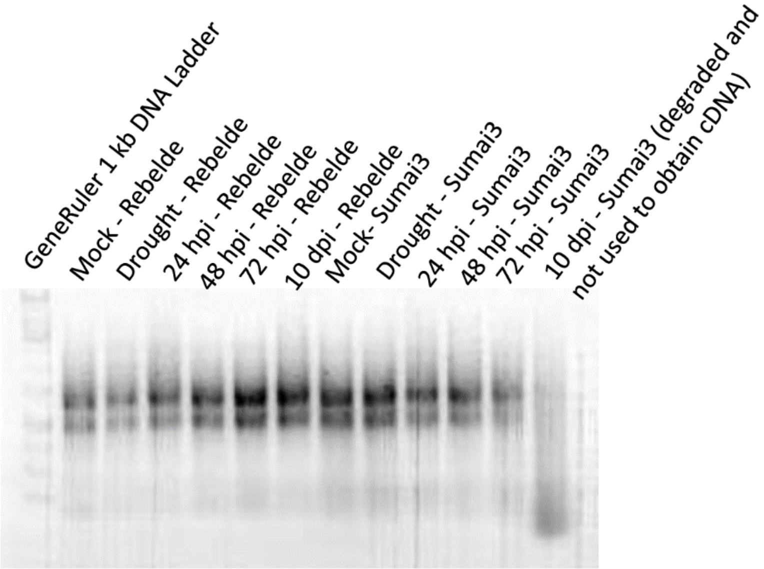

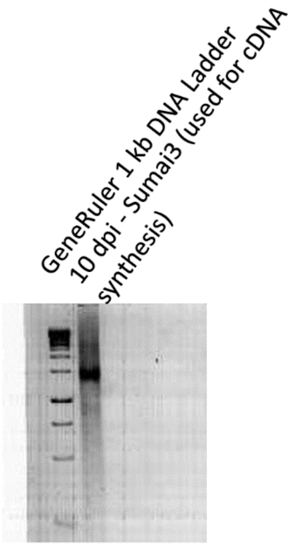

Original pictures for the S4 Figure (A and C).
Only the edges of the gel were cropped.

The pictures belong to two different gels.
